# Review research progress, critical reflections and future prospects of digital care for chronic diseases

**DOI:** 10.3389/fpubh.2026.1968554

**Published:** 2026-10-13

**Authors:** Yingchao Zhou, Ruizhe Cao, Yanwei Su

**Affiliations:** School of Nursing, Tongji Medical College, Huazhong University of Science and Technology, Wuhan, China

**Keywords:** chronic disease management, digital care, evidence-based nursing, health equity, telemedicine

## Abstract

The global prevalence of chronic diseases is becoming increasingly severe, and traditional care models are confronted with multiple challenges, including uneven distribution of medical resources, insufficient continuity of care, and inadequate patient self-management capacity. The rapid iteration of digital technologies and the catalyst of the COVID-19 pandemic have driven chronic disease care into a new phase of digital transformation. Based on relevant studies published from 2021 to 2026, this paper structures its analysis around a core logical framework: evidence-based evidence, care models, population adaptability and practical implementation. It systematically reviews the core technical systems and innovative characteristics of care models in digital chronic disease care, deeply analyzes the adaptive differences across diverse populations and disease types, and conducts critical reflections from the perspectives of evidence quality, health equity, clinical applicability, and homogeneity in intervention design. Finally, it puts forward future development directions centered on precision, localization, equity, and integration. The study reveals that digital chronic disease care has formed four core technological systems in application: intelligent sensing, digital behavioral intervention, immersive technology, and artificial intelligence (AI) and big data. Care models demonstrate innovative trends of nurse leadership, multi-dimensional collaboration, and caregiver empowerment. Nevertheless, prominent issues persist, such as insufficient high-quality evidence, a striking digital divide, difficulties in clinical integration, and a lack of personalized design. In the future, deep integration of technological innovation with clinical demands, enhanced targeted interventions for vulnerable populations, implementation of localized long-term studies, and improvement of policy and ethical guarantee systems are required. These efforts will promote the shift of digital chronic disease care from technology-driven to evidence-driven and demand-driven models, ultimately improving the overall efficiency of chronic disease management and health equity.

## 1 Introduction

### 1.1 Global status of chronic disease prevalence and key pain points of traditional care models

Chronic non-communicable diseases (NCDs) have emerged as a central challenge in global public health. According to data from the Global Burden of Disease Study 2019 (GBD 2019), there were 23.6 million new incident cases of 29 categories of malignant neoplasms worldwide, resulting in 10.0 million deaths and 250 million disability-adjusted life-years (DALYs) lost. From 2010 to 2019, these three indicators increased by 26.3, 20.9, and 16.0% respectively, placing cancer as the second leading cause of global disease burden (1).

Beyond cancer, other chronic conditions such as cardiovascular diseases, diabetes mellitus, and chronic obstructive pulmonary disease (COPD) also show a high and growing prevalence. Taking diabetes as an example, the global number of adults living with diabetes has exceeded 400 million and continues to rise (2).

The chronicity, complexity and high recurrence of chronic diseases pose severe challenges to the traditional nursing model. On the one hand, medical resources are overly concentrated on acute-phase treatment, whereas community and home-based care resources are in short supply, resulting in disrupted continuity of care for patients after hospital discharge. On the other hand, patients present weak self-management capacity and lack access to scientific health monitoring, medication guidance and lifestyle interventions, which leads to repeated deterioration of conditions and an increased medical burden. Furthermore, the uneven allocation of medical resources across regions and population groups further aggravates inequities in chronic disease management. During the COVID-19 pandemic, chronic disease diagnosis and treatment services in sub-Saharan Africa were severely interrupted, marked by prominent problems such as reduced outpatient volume, fewer follow-up visits, and delayed diagnosis and treatment (3).

The numerous shortcomings of the traditional chronic disease care model urgently require novel technical means and service models for resolution. The rapid development of digital technologies, including the Internet of Things (IoT) and artificial intelligence (AI), has furnished core technical support for the digital transformation of chronic disease care, representing a vital direction for breaking through the aforementioned dilemmas.

### 1.2 Developmental background of digital technology-enabled chronic disease care

The rapid advancement of digital technologies has provided core support for the transformation of chronic disease care. The deep integration of the Internet of Things (IoT), artificial intelligence (AI), virtual reality (VR), augmented reality (AR), and other technologies with nursing scenarios has spawned novel care models such as remote monitoring, digital behavioral intervention, and intelligent decision support. Tools including wearable devices, implantable electronic devices, and remote monitoring platforms enable real-time collection and remote transmission of health data. For instance, artificial intelligence and big data technologies have driven the intelligent upgrading of health management, spanning from data acquisition to risk early warning and personalized intervention (4).

The COVID-19 pandemic has further accelerated the widespread adoption of digital care. Virtual visits, remote consultations and other approaches have effectively alleviated difficulties in patient access to medical care during the pandemic, serving as a critical strategy to maintain the continuity of chronic disease care (5). For example, remote monitoring for patients with heart failure was rapidly implemented during the pandemic, enabling early identification of clinical deterioration and effectively reducing hospitalization risk (5). Similarly, patients with chronic obstructive pulmonary disease (COPD) maintained consistent rehabilitation training via virtual reality-assisted pulmonary rehabilitation despite limited hospital access, leading to improvements in pulmonary function and exercise tolerance (6).

### 1.3 Research evolution of digital care for chronic diseases and limitations of existing reviews

In recent years, research in the field of digital care for chronic diseases has grown exponentially, covering multiple dimensions such as technological development, intervention efficacy validation, and exploration of care delivery models (7). Existing studies have confirmed that digital care holds potential value in improving patients’ clinical indicators, enhancing self-management capabilities, and reducing the consumption of medical resources. Nevertheless, current review studies suffer from notable limitations. First, most reviews adopt a single-dimensional research focus, concentrating on a specific type of digital tool (e.g., wearable devices, mobile health applications) or a particular chronic disease (e.g., diabetes, heart failure), while lacking integrated analyses across technologies, diseases, and populations (8). Second, they demonstrate insufficient critical evaluation, often consisting of simple literature aggregation and summaries of positive findings, with inadequate attention to issues such as flaws in research design, variations in evidence quality, and implementation barriers (9). Third, they are deficient in practical applicability, neglecting the adaptability of digital care in regions with different economic development levels and among populations with varying levels of digital literacy, and providing insufficient discussion on health equity and pathways to clinical translation. Fourth, they lack a systematic framework, failing to form a complete logical chain of technology–model–population–implementation, which limits their ability to guide clinical practice and policy formulation (10).

### 1.4 Research aims, scope and innovative perspectives of this review

Prior to presenting the study aims, we first clarify two core concepts used throughout this manuscript:

Digital care for chronic diseases: A broad umbrella term referring to the integration of digital technologies (e.g., remote monitoring, AI, immersive technologies) into chronic disease prevention, diagnosis, treatment, rehabilitation and self-management, delivered by multidisciplinary health teams and supporting patient self-care. It covers all technology-enabled care activities across the entire chronic disease care continuum.

Digital nursing: A core subdomain of digital care that specifically refers to nursing practice delivered or supported by digital technologies, including nurse-led remote monitoring, digital health education, virtual care coordination, and technology-supported self-management guidance. It represents the nursing-specific implementation of digital care principles, with nurses as the core care deliverers.

This manuscript centers on the broader topic of digital care for chronic diseases (consistent with the title), with a particular focus on nursing-led practice models and nursing-related evidence, which explains the alternating use of both terms in context.

This review aims to construct a comprehensive and critical research framework for digital care in chronic diseases, based on relevant studies published between 2021 and 2026, including systematic reviews, randomized controlled trials, cohort studies, qualitative studies, and other research designs. The scope of this review covers a variety of chronic conditions such as cardiovascular diseases, diabetes mellitus, respiratory diseases, cancer, and rare diseases. It involves core digital technologies including intelligent sensing, digital behavioral intervention, immersive technologies, artificial intelligence, and big data analytics, and encompasses populations of different ages, ethnicities, and socioeconomic statuses.

The innovative perspectives of this review are mainly reflected in three aspects. First, innovation in logical framework: taking the core logical chain of evidence-based evidence – care model – population adaptation – practical implementation, this review breaks the limitations of traditional single-dimensional reviews and achieves cross-dimensional and multi-level integrated analysis. Second, innovation in critical perspective: it not only summarizes the positive effects of digital care, but also focuses on key issues such as evidence quality, health equity, and clinical translation, and deeply analyzes the limitations and inconsistencies of existing research. Third, innovation in future directions: based on critical reflection, this review proposes targeted strategies for technological optimization, model innovation, population adaptation, and policy support, providing clear guidance for clinical practice, research design, and policy formulation. Figure 1 illustrates the overarching analytical framework of this review, which is structured along the core logical chain of research background, core technical system, innovative care models, adaptability-related critical reflections, and future directions, with policy and ethical safeguards as the foundational support throughout the entire framework.

**Figure 1:**
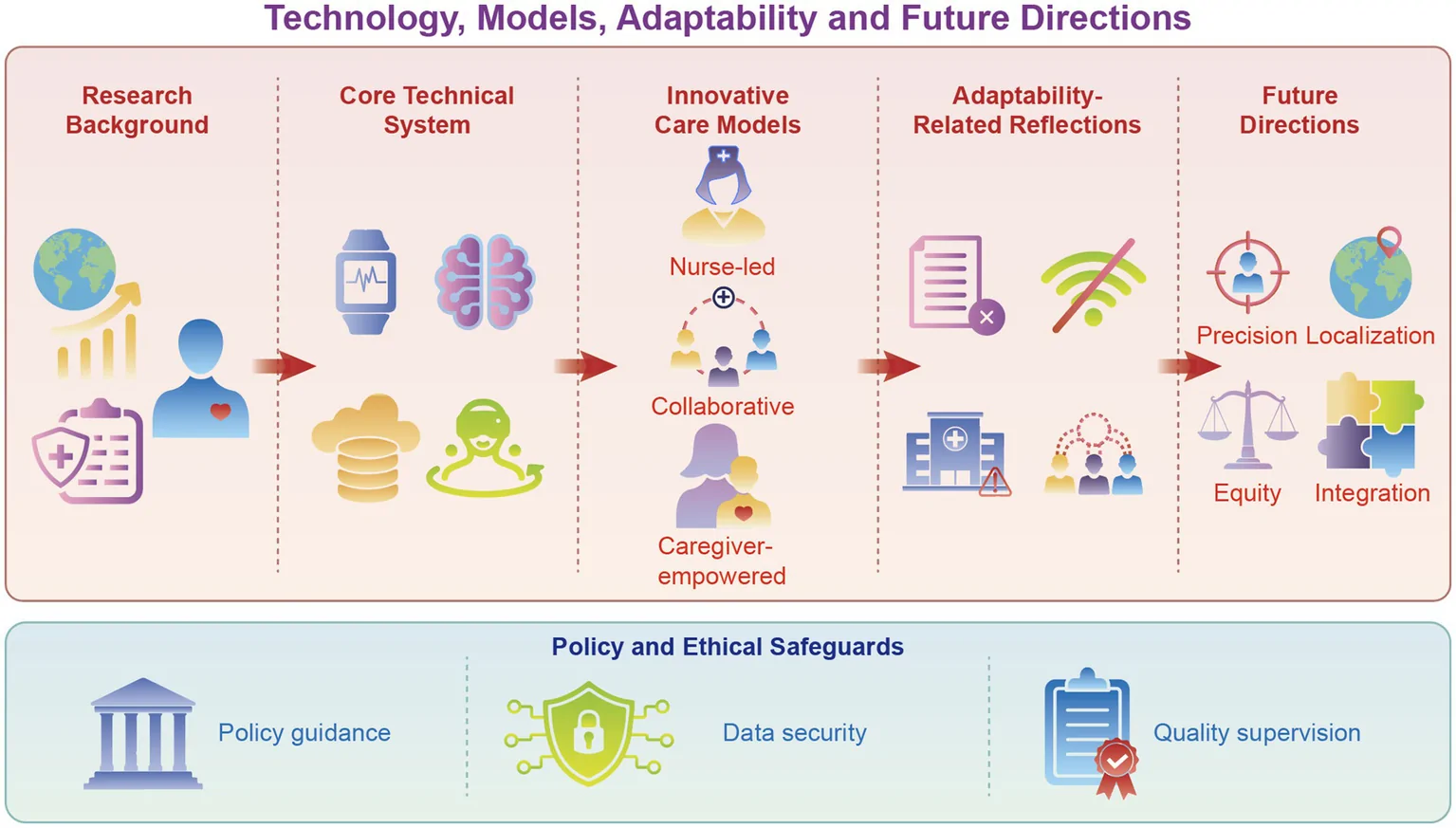
This figure illustrates the overarching analytical framework of this narrative review, structured along the core logical chain of research background, core technical system, innovative care models, adaptability-related critical reflections, and future development directions. Policy and ethical safeguards (covering policy guidance, data security, and quality supervision) serve as the foundational support running through the entire research framework. Four strategic pillars for future development—precision, localization, equity, and integration—are proposed to guide the high-quality evolution of digital chronic disease care.

## 2 Method

### 2.1 Methods

#### 2.1.1 Study design and database selection

This narrative review was conducted in accordance with the Scale for the Assessment of Narrative Review Articles (SANRA) guidelines (a widely accepted framework for narrative review reporting) and standard conventions for chronic disease nursing research. PubMed was selected as the primary database for this review after full consideration of alternative databases including Embase, CINAHL and Scopus. This decision was driven by three key rationales: first, PubMed indexes the vast majority of high-quality, peer-reviewed international studies in the fields of chronic disease management and digital nursing care, ensuring core evidence coverage; second, its standardized MeSH indexing system enables consistent and reproducible search strategies for narrative synthesis; third, restricting to a single database maintains consistent search filters and eligibility thresholds across all retrieved records, avoiding cross-database heterogeneity in study indexing and publication type classification. We acknowledge that this single-database approach may omit studies indexed exclusively in other databases, which may introduce database selection bias and limit the comprehensiveness of the evidence base.

### 2.2 Full search strategy

The search strategy combined both Medical Subject Headings (MeSH) terms and free-text keywords, and the literature search was conducted on 25 March 2026. The full search string combined terms with Boolean operators AND/OR as follows:

(“Chronic Disease”[MeSH Terms] OR “Noncommunicable Diseases”[MeSH Terms] OR chronic disease*) AND ((“Nursing Care”[MeSH Terms] AND (“Digital Health”[MeSH Terms] OR “Telemedicine”[MeSH Terms])) OR digital nursing OR telenursing OR virtual nursing) AND “humans”[MeSH Terms].

Two tiers of pre-screening filters were applied directly in the PubMed search system. First, publication date was restricted to the last 5 years (March 2021 to March 2026), and only free full-text publications were included, yielding an initial set of 405 records. This 5-year time frame was selected to capture the most recent technological iterations, real-world implementation experiences and post-pandemic evolution of digital health technologies in chronic disease care, ensuring the review reflects state-of-the-art care models and up-to-date evidence. The free full-text restriction was applied to enable full-text quality appraisal, detailed data extraction and in-depth narrative synthesis of all included studies, rather than reliance on abstract-only information. We acknowledge that this restriction may introduce open-access selection bias by excluding subscription-only publications, and we have addressed this limitation explicitly in the Limitations section. Foundational theoretical studies published before 2021 are cited as conceptual background but not included in the formal evidence synthesis.

Second, article type filters were applied to restrict results to peer-reviewed research and review publications of relevant study designs, including: Clinical Study, Clinical Trial, Meta-Analysis, Practice Guideline, Randomized Controlled Trial, Review, and Systematic Review. After applying article type filters, 179 records remained for formal title and abstract screening.

No additional handsearching of reference lists was performed to maintain scope consistency and avoid introducing unstandardized literature sources.

### 2.3 Study selection process

As the review was based on a single database search, no cross-database duplicate records were identified. Two independent reviewers screened the titles, abstracts, and full texts of the 179 retrieved records sequentially. Disagreements on eligibility judgment or data extraction were resolved by discussion between the two reviewers; if consensus could not be reached, a third senior reviewer made the final arbitration. The study selection process followed the Preferred Reporting Items for Systematic Reviews and Meta-Analyses (PRISMA) 2020 guidelines, with the number of records at each screening stage documented systematically.

Overall, 179 records entered the title and abstract screening phase, among which 36 were excluded for ineligibility. Full texts of 143 records were further assessed for eligibility, among which 59 articles were excluded, and 84 studies were finally included in the narrative synthesis.

### 2.4 Eligibility criteria

Eligible studies met the following criteria:

1. Participants were adult (≥18 years) patients diagnosed with one or more chronic diseases (including but not limited to cardiovascular diseases, diabetes mellitus, chronic obstructive pulmonary disease, chronic kidney disease, and malignant tumors). Studies focusing on pediatric and adolescent chronic disease populations were excluded from the formal evidence synthesis, and are only discussed in the Population Adaptability section as comparative context to highlight age-related differences in digital care applicability.
2. Investigated the application, effects, implementation barriers, or theoretical frameworks of digital health-assisted nursing care for chronic disease management, covering modalities including telemedicine nursing, digital nursing, virtual nursing, and technology-supported chronic care models;
3. Study designs consistent with the applied PubMed filters: clinical studies, clinical trials, randomized controlled trials, meta-analyses, systematic reviews, narrative reviews, and practice guidelines;
4. Full-text articles available as free full text on the PubMed platform;
5. Conducted in clinical, community, or home-based care settings.

Original empirical studies constitute the core evidence base of this review; review articles and practice guidelines are included only for auxiliary purposes such as research landscape mapping and cross-study comparison, and do not serve as direct evidence for core conclusions.

Exclusion criteria included non-free full-text publications, conference abstracts, letters, editorials, case reports, study protocols, studies focusing on pediatric chronic disease populations or professional caregiver training only, as well as studies that failed to meet the thematic eligibility requirements. A small number of out-of-scope publications (including pediatric and adolescent studies) were only cited as background contextual references for cross-population comparison and did not participate in the formal evidence synthesis.

### 2.5 Quality appraisal

Prior to narrative synthesis, the methodological quality of included studies was appraised using the Joanna Briggs Institute (JBI) critical appraisal checklists corresponding to each study design, to inform the grading of evidence strength and the interpretation of findings. All eligible studies meeting the inclusion criteria were included in the final narrative synthesis, with quality appraisal results used to contextualize the reliability and applicability of reported findings.

### 2.6 Data extraction and narrative synthesis

Data were extracted using a standardized data extraction form, which captured basic study characteristics (authors, publication year, country, study design), participant demographics, digital nursing intervention details, core outcome indicators, and key research findings. Extracted data were synthesized narratively around predefined core thematic domains.

No quantitative meta-analysis was performed due to the substantial heterogeneity in study designs, digital intervention modalities, and outcome measurement tools across included studies. Included studies were categorized by study type to clarify the evidence hierarchy: original empirical studies (including randomized controlled trials, clinical trials, and observational clinical studies) formed the core evidence base for synthesizing application effects, implementation mechanisms, and practical challenges of digital nursing for chronic diseases; systematic reviews, narrative reviews, and practice guidelines were used only to contextualize the research background and compare findings with prior evidence syntheses.

## 3 Core technical system and evidence-based application characteristics of digital care for chronic diseases

The technological system of digital care for chronic diseases has formed a multi-dimensional and full-coverage development landscape. From health data collection and risk prediction to behavioral intervention and intelligent decision-making, various digital technologies are deeply integrated with nursing scenarios. Nevertheless, their evidence-based effects vary considerably, and the efficiency of clinical translation needs to be further improved. The technological system of digital chronic disease care has formed a complete closed-loop architecture spanning data collection, secure transmission, intelligent analysis, and intervention execution, as visualized in Figure 2.

**Figure 2:**
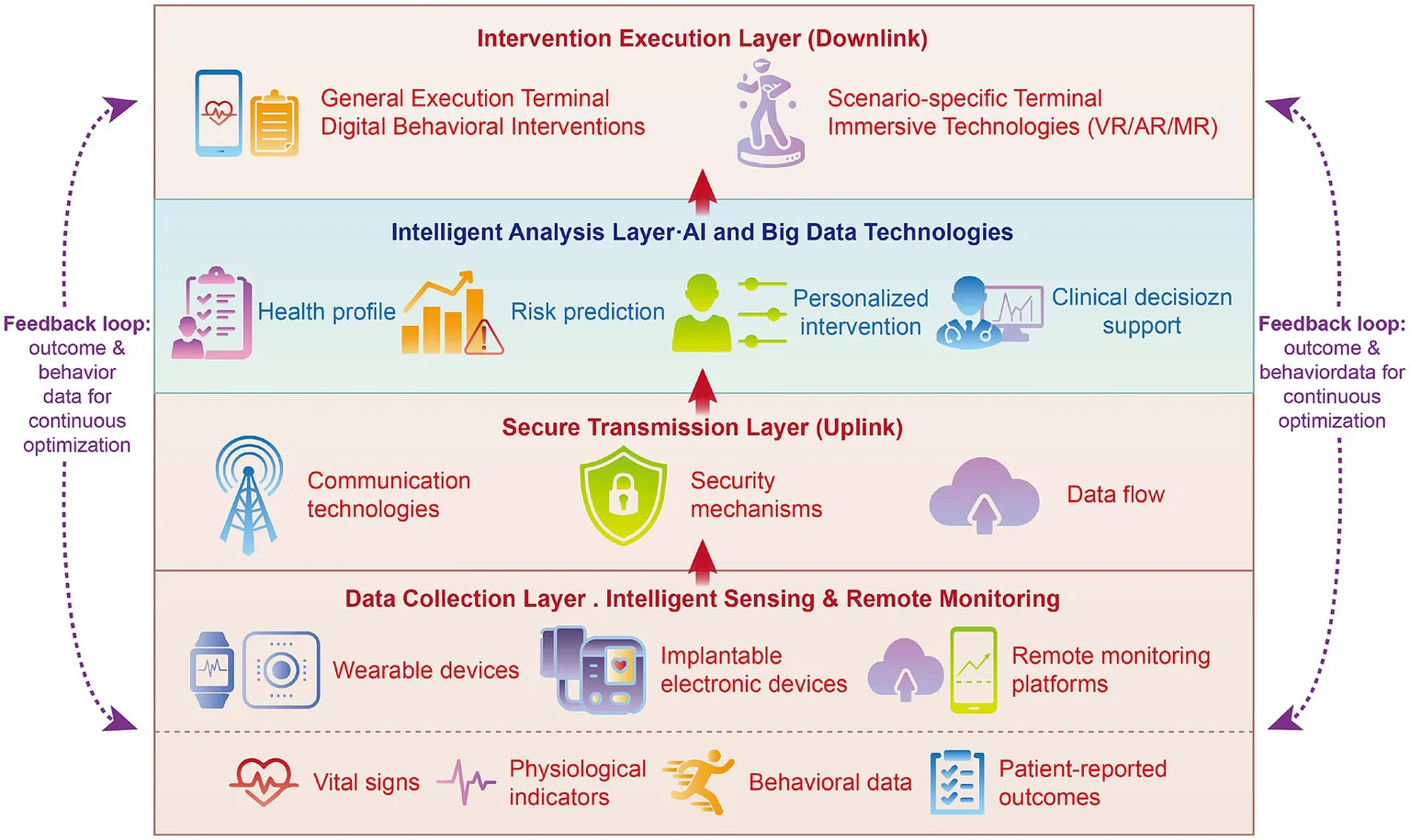
This figure depicts the four-layer closed-loop technical architecture underpinning digital chronic disease care. From bottom to top: (1) the data collection layer, which acquires vital signs, physiological indicators, behavioral data, and patient-reported outcomes via wearable devices, implantable electronic devices, and remote monitoring platforms; (2) the secure transmission layer, which enables uplink data flow through communication technologies and standardized security mechanisms; (3) the intelligent analysis layer, which leverages AI and big data technologies to deliver health profiling, risk prediction, personalized intervention, and clinical decision support; (4) the intervention execution layer, which delivers downlink care services through general terminals for digital behavioral interventions and scenario-specific terminals for immersive technologies (VR/AR/MR). Bilateral feedback loops support continuous system optimization based on real-world outcome and behavioral data.

### 3.1 Intelligent sensing and remote monitoring technologies: from data acquisition to risk early warning

Intelligent sensing and remote monitoring technologies constitute the foundation of digital care for chronic diseases. Through tools such as wearable devices, implantable electronic devices, and remote monitoring platforms, they enable real-time collection and remote transmission of vital signs, physiological indicators, and behavioral data, providing data support for early risk identification and precise intervention (5).

In terms of technical types and application scenarios, wearable devices have established a multi-parameter monitoring system, including multi-parameter sensor patches, the ReDS™ vest (for heart failure monitoring), smart watches, and similar devices, which can monitor a variety of indicators such as heart rate, blood pressure, oxygen saturation, and physical activity (5). Cardiac implantable electronic devices (including ICD, CRT-D, etc.) and remote hemodynamic monitoring devices (such as CardioMEMSTM) are designed for patients with cardiovascular diseases, enabling continuous monitoring of cardiac function and hemodynamic parameters (5). In addition, remote monitoring platforms (e.g., the Dignio Connected Care platform), integrated with mobile applications, provide functions such as patient-reported outcome measures, asynchronous communication, and personalized reminders, thereby realizing multi-dimensional data integration and doctor-patient interaction (11).

Stratified analysis of evidence-based effects reveals substantial variations in the clinical value of different monitoring technologies. Several technologies have gained robust evidence support; for instance, the CardioMEMSTM remote hemodynamic monitoring system enables early detection of clinical deterioration in patients with heart failure and significantly reduces hospitalization risk (5). However, the efficacy of certain technologies remains controversial. For example, the OptiVol remote monitoring strategy has not been shown to improve clinical outcomes in patients with heart failure (5). Among studies investigating digital literacy training for older adults with cardiovascular disease, only two trials confirmed that such training improved both technology utilization and health outcomes, whereas most studies merely verified the necessity of training without demonstrating clear clinical benefits (12). Furthermore, numerous monitoring technologies are still in the pilot validation phase. Smart inhalers used for medication adherence monitoring in patients with asthma and COPD can improve adherence rates but lack support from large-sample long-term clinical data (13). Similarly, the clinical effectiveness of salt intake monitors used to track dietary sodium consumption in patients with hypertension requires further validation (14).

Intelligent sensing and remote monitoring technologies face the core challenge of disconnection between data collection and clinical translation. Most technologies only achieve the collection and transmission of health data, yet fail to establish a closed-loop management system of data collection-risk early warning-intervention delivery-outcome feedback (10). Beyond the core issue of data translation, the practical implementation of commercial digital devices in clinical settings has clear application boundaries due to differences in target populations and supporting infrastructure. The realization of their clinical value relies on three specific prerequisites. First, they are only suitable for chronic disease patients with adequate digital health literacy who can operate smart devices proficiently, and have limited adaptability for individuals with poor digital literacy, cognitive impairment, or inability to use devices independently. Second, effective device use and reliable data collection by patients require nurse-led specialized training and ongoing support. Third, device application must be restricted to predefined scenarios and depend on consistent patient adherence to wearing protocols to ensure data integrity (14).

Furthermore, inherent technical limitations hinder clinical adoption. Commercial devices vary considerably in measurement accuracy (15); some wearable devices show poor adaptability among older adults and patients with multimorbidity. The comfort and tolerability of sensor wearing need to be improved (16).

### 3.2 Digital behavioral interventions and cognitive therapies: from health education to personalized behavior modification

Digital behavioral interventions focus on improving patients’ self-management capabilities. Supported by behavior change theories such as the Information-Motivation-Behavioral Skills (IMB) model, these interventions deliver health education and medication guidance via mobile health tools and online health counseling (17).

The core application forms include the following: First, mobile health (mHealth) applications. For example, the WeChat official account ‘Diabetes Assistant’ delivers animated videos to provide medication guidance and self-management education for patients with diabetes, based on the Information-Motivation-Behavioral Skills (IMB) model (17). Second, digital cognitive behavioral therapy (CBT). Examples include online self-management programs for patients with pancreatitis-related pain (18), and digital CBT interventions delivered via Facebook Messenger for those with sickle cell disease-related pain (19). Third, gamified interventions. For instance, a gamified digital pursed-lip breathing training program based on WeChat Mini Programs uses rankings, daily tasks, and social community modules to improve rehabilitation adherence among patients with COPD (20). Fourth, online health coaching. For example, nurses conduct virtual health literacy classrooms via WhatsApp, employing simple language, illustrations, and the teach-back method to enhance treatment adherence among patients with type 2 diabetes (21).

Evidence-based analysis demonstrates that digital behavioral interventions offer certain advantages in improving short-term clinical outcomes. Regarding medication adherence, mobile video interventions significantly improve adherence among older adults with type 2 diabetes; adherence scores in the intervention group were significantly higher than those in the control group both post-intervention and at the 3-month follow-up (17). Following WhatsApp virtual classroom interventions, treatment adherence scores of patients with type 2 diabetes increased significantly, with a statistically significant between-group difference (*p* < 0.001) (21).

In pain management, digital CBT reduces pain interference and improves quality of life in patients with sickle cell disease. For patients with recurrent acute pancreatitis, digital CBT interventions show promise in reducing both pain interference and pain severity (18).

In terms of lifestyle modification, digital interventions significantly reduce total daily sedentary time by 30.80 min and the proportion of sedentary behavior by 4.65% among patients with chronic diseases (22). These findings confirm that digital behavioral interventions effectively reduce sedentary behavior in this population. Moreover, exercise interventions integrated with wearable devices can further translate reduced sedentary behavior into increased voluntary physical activity, thereby optimizing lifestyle across multiple dimensions. Additionally, wearable-supported exercise programs significantly increase moderate-to-vigorous physical activity and daily step counts in cancer survivors (23).

However, prominent issues exist regarding the long-term effectiveness and sustained engagement of digital behavioral interventions. Most studies indicate that the beneficial effects of digital interventions are significant in the short term (3–6 months) but diminish during long-term follow-up (over 1 year), accompanied by a continuous decline in patient engagement. For instance, community-based digital interventions for type 2 diabetes can expand coverage but suffer from insufficient sustained participation (2). In digital CBT interventions, patients show low engagement with digital modules and rely heavily on support from health coaches (19).

Furthermore, intervention effects demonstrate substantial heterogeneity, influenced by factors such as the design of digital literacy training (DLT) and technological adaptability (e.g., user-friendliness). Low digital literacy does not necessarily predict poor intervention outcomes; instead, targeted supporting measures — including device provision, in-person training, printed manuals, and hotline support — are required to remove usage barriers. Overall, older adults with cardiovascular disease can benefit from such interventions, with favorable technology acceptance and utilization outcomes (12).

In terms of innovative applications, optimized intervention designs grounded in behavior change theories have emerged as a research hotspot. For example, mobile video interventions based on the Information-Motivation-Behavioral Skills (IMB) model focus not only on health knowledge delivery but also on motivation enhancement and behavioral skills training, which significantly improve medication-related knowledge, attitudes, and behaviors among patients with diabetes (17). Digital gamified pursed-lip breathing training developed within the Behavior Change Wheel framework integrates multiple behavior change techniques such as goal setting, feedback, and social support, thereby enhancing intervention attractiveness and adherence (20).

### 3.3 Immersive technologies (VR/AR/MR): from symptom relief to full-cycle rehabilitation care

Immersive technologies such as virtual reality (VR), augmented reality (AR), and mixed reality (MR) create virtual environments to deliver diversified care services for patients with chronic diseases, including rehabilitation training, pain management, and psychological interventions. Their core advantage lies in enhancing the attractiveness and immersion of interventions, thereby boosting patient engagement (24).

The application dimensions mainly cover eight areas: medical procedure training, healthcare professional training, substance use disorder treatment, cognitive rehabilitation, physical rehabilitation, exergaming and biofeedback, navigation and spatial therapy, and pain, stress and anxiety management (24).

In chronic disease care, the most widespread applications are in physical rehabilitation, pain management and psychological interventions. For instance, VR-assisted pulmonary rehabilitation for patients with COPD improves pulmonary function, exercise tolerance and dyspnea through virtual scenario-based interactive training (6).

Evidence-based characteristics indicate that immersive technology-assisted rehabilitation demonstrates clear short-term benefits, yet its long-term efficacy and population adaptability remain to be verified. Among patients with COPD, VR combined with pulmonary rehabilitation significantly improves forced expiratory volume in one second (FEV1), forced vital capacity (FVC), six-minute walking distance, and relieves dyspnea compared with pulmonary rehabilitation alone, with optimal effects observed at 5–12 weeks of intervention (6). In chronic pain management, VR exhibits promising potential to relieve chronic back pain, complex regional pain syndrome and other chronic pain conditions; however, existing studies are highly heterogeneous in pain categories, intervention designs and research methodologies, with great variability in the degree of VR immersion across trials and a large number of interventions lacking interactivity and adopting passive virtual environment modes (25). A study conducted in Turkey reported that viewing nature videos via Oculus Quest 2 VR headsets did not significantly reduce pain or anxiety in patients with chronic lower extremity wounds, which may be attributed to inadequate immersion caused by first-time use and the absence of interactive content (26). In cognitive rehabilitation, VR-based cognitive training for patients with Alzheimer’s disease improves cognitive and spatial functions, yet long-term follow-up data are lacking (24).

In existing studies, older adults with chronic lower extremity wounds show poor VR immersion, mainly due to advanced age and first-time exposure to VR devices. Only a small proportion of patients (approximately 11%) experienced mild discomfort, including headache, eye strain, blurred vision, or slight general malaise, with no reports of motion sickness (26).

In addition, standardization of VR interventions remains a prominent issue. Substantial variations exist across studies in terms of devices used (e.g., BioMaster, Wii, Xbox Kinect), intervention duration, and virtual environment design, which hinder evidence synthesis and widespread implementation (27).

### 3.4 AI and big data technologies: from precision assessment to intelligent decision support

AI and big data technologies enable precise assessment, risk prediction, personalized intervention, and intelligent decision support for chronic diseases by integrating multi-source health data, and have become a core innovative direction in digital chronic care (10).

Core applications span four major areas: First, health profile construction. Based on electronic health records, wearable device data, sensor data, etc., four types of health profiles are established-diagnostic, prognostic, monitoring, and recommendation-providing a foundation for personalized management (28). Second, risk prediction models. Machine learning algorithms are used to predict the onset risk, deterioration risk, and complication risk of chronic diseases such as hypertension (29). Third, personalized interventions. Examples include the AI-based Health Education and Advice Learning System (AI-HEALS) rooted in traditional Chinese medicine constitution theory, which delivers tailored health guidance for patients with multimorbidity. Fourth, intelligent decision support (30). For instance, AI wound assessment tools employ image recognition to automatically perform wound segmentation and tissue classification, assisting clinical decision-making (31). Research progress indicates that the application potential of AI and big data technologies in chronic disease care has been preliminarily verified. In terms of health profiling, recommendation-oriented profiles perform best:17.78% of studies meet all three 3 V criteria of big data (Volume, Velocity, Variety), 30.34% complete external validation, yet only 10.11% satisfy both standards simultaneously, suggesting overall quality needs improvement (28). In risk prediction, AI can improve cuffless blood pressure measurement in hypertension, enhance risk prediction and medication individualization, and support patient self-management, but faces issues such as data bias and insufficient validation (29). In personalized interventions, the TCM constitution-adapted AI-HEALS system delivers interventions via WeChat Mini Programs, with ongoing trials planned to verify its effects on improving systolic blood pressure, blood glucose, lipids, and other indicators in patients with multimorbidity (30). In intelligent decision support, an AI wound assessment tool developed in Switzerland achieved a DICE score of 92% and IOU of 85% for wound segmentation, and an average DICE of 78% for tissue classification. After quantitative optimization, mobile inference takes only 0.3 s with a mere 0.3% performance reduction (31).

AI and big data technologies face core challenges including algorithmic bias, insufficient external validation, and a lack of localized training. Most algorithms are developed using data from high-income countries and lack localized training for low- and middle-income countries and vulnerable populations (e.g., ethnically diverse groups and older adults), leading to algorithmic bias and limited generalizability (32). Among health profiling studies, only 30.34% completed external validation, and merely 10.11% satisfied both the 3 V criteria and external validation, resulting in insufficient evidence reliability (28). The clinical translation efficiency of AI tools remains low: most are limited to laboratory research or small-sample pilot studies, lacking large-scale real-world evidence to verify their effectiveness and safety (10). In addition, prominent issues surround data privacy and security. Patient concerns regarding privacy hinder data sharing and acceptance of digital tools, and the data security and ethical implications of AI algorithms have not been adequately addressed (10). In summary, the four core technical systems of digital chronic care each demonstrate distinct application characteristics and evidence-based value, yet all face varying challenges in clinical translation and population adaptation. The implementation effectiveness of these technologies is also closely associated with innovations in the delivery entities and care models of chronic disease management. The following section further analyzes the characteristics of care models in digital chronic care.

## 4 Implementers and innovative care models in digital chronic disease care

The application of digital technologies has not only transformed the technical tools of chronic disease care but also restructured its implementers and care delivery models. The traditional model centered solely on healthcare professionals is gradually shifting toward a diversified model characterized by nurse-led, multi-dimensional collaboration, and caregiver empowerment. These models differ substantially in evidence-based outcomes, applicable scenarios, and implementation barriers. Three dominant innovative care models have been developed in digital chronic disease care practice: the nurse-led model, the multi-dimensional collaborative model, and the caregiver-empowered model. Figure 3 summarizes the practice framework of the three models across four core dimensions.

**Figure 3:**
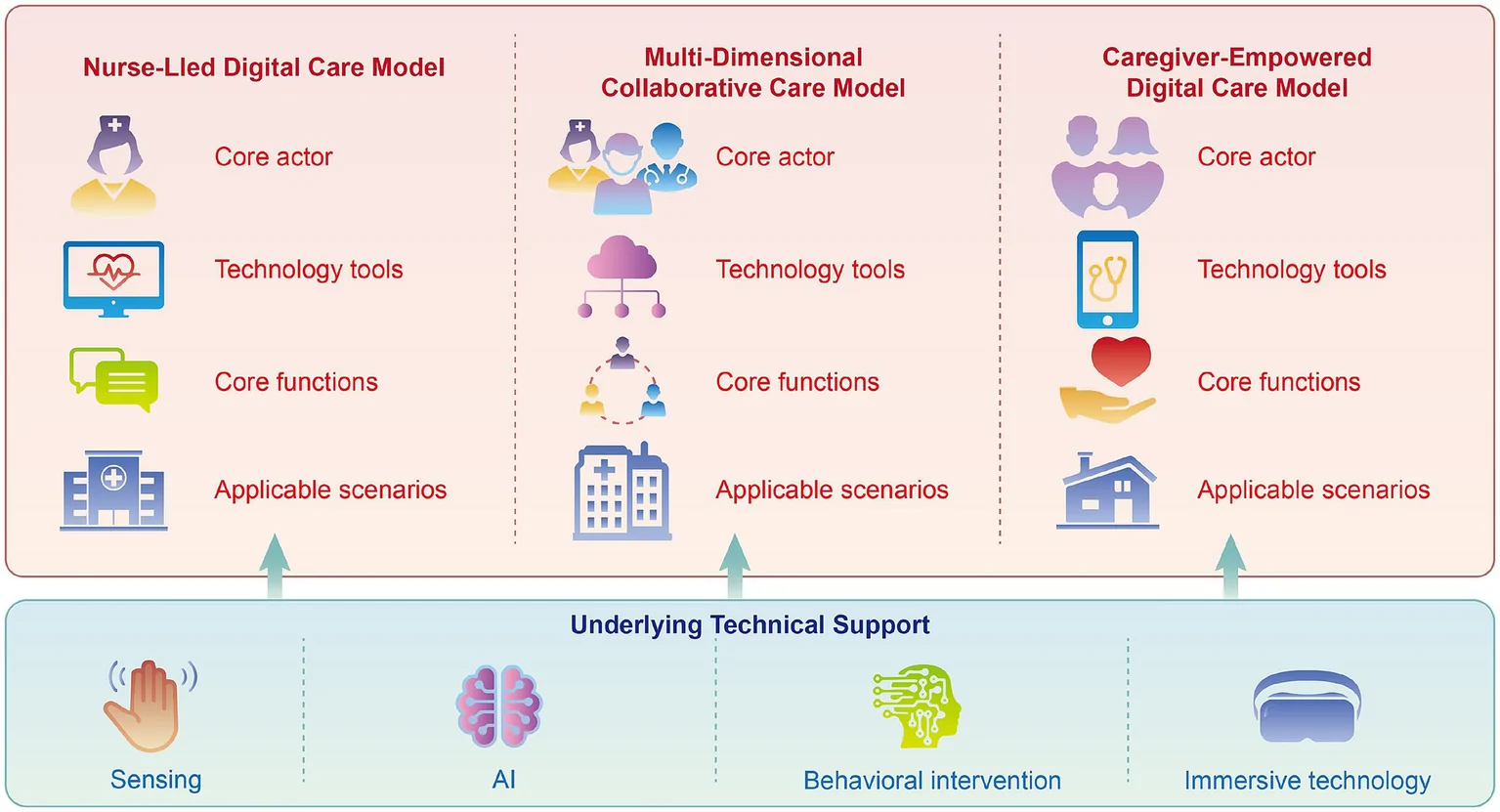
This figure summarizes the practice framework of three dominant digital chronic disease care models: the nurse-led digital care model, the multi-dimensional collaborative care model, and the caregiver-empowered digital care model. Each model is delineated across four consistent dimensions: core actor, technology tools, core functions, and applicable scenarios. Four categories of underlying technical support—sensing technology, artificial intelligence, behavioral intervention technology, and immersive technology—constitute the shared technical foundation enabling the implementation of all three care models.

### 4.1 Nurse-led digital care models: core roles and practical boundaries

As the core implementers of chronic disease care, nurses undertake multiple key roles in digital care, including care coordination, remote monitoring, chronic disease management, and triage assessment. The nurse-led digital care model has become an important complementary approach to chronic disease management.

Nurses perform a range of core roles and adopt multiple delivery modalities in primary care virtual services, predominantly providing care via telephone with video, messaging and email as supplementary approaches, and centering their practice on care coordination, chronic disease management, and triage and assessment.

In specialized chronic disease management, nurse-led remote monitoring and case management yield significant effects. For example, in a one-arm intervention study conducted in Thailand, nurses implemented case management for noncommunicable diseases using digital tools including smart watches, dedicated health applications, and salt intake monitors, combined with face-to-face health education, real-time health data surveillance, SKT meditation for stress reduction, and self-management manuals and diaries. This intervention is expected to improve patients’ self-management behaviors and vascular health-related clinical outcomes (14).

In teleconsultations, nurses provide care for patients with chronic conditions such as cancer, asthma, COPD, heart failure, and diabetes through real-time video or telephone consultations, supplemented by medical record review and peer support, thereby enhancing healthcare accessibility and patients’ self-management capacity (33).

Evidence-based analysis demonstrates that nurse-led digital interventions offer clear benefits in improving clinical indicators, reducing healthcare resource utilization, and enhancing care satisfaction. A review of 40 randomized controlled trials across 16 countries found that nurse-led digital interventions (delivered via websites, mobile applications, novel monitoring systems, and other tools) significantly improved patients’ self-management abilities and clinical outcomes, reduced healthcare resource consumption, and increased care satisfaction, with mobile apps showing the most pronounced effects (7).

Among overweight and obese adults receiving nurse-led telecare interventions, average weight loss reached 2.59 kg, BMI decreased by 1.05 kg/m^2^, and waist circumference was reduced by 2.52 cm, indicating significant short-term benefits in weight reduction and lifestyle modification (34).

In patients with multimorbidity, nurse-led interventions improved care quality, medication adherence, self-management, and self-efficacy; optimized physiological indicators such as blood pressure and blood glucose; and reduced readmission and mortality rates (35).

Nurse-led digital care models are constrained by multiple limitations, including digital literacy, training resources, and practical boundaries. Uneven digital literacy among nurses persists, with some lacking proficiency in operating digital tools, interpreting health data, and delivering remote interventions, which compromises intervention effectiveness. Insufficient nursing training and equipment represent major implementation barriers; most studies do not clarify how nurse qualifications influence outcomes, and standardized digital care training frameworks remain lacking. Although teleconsultations improve patient accessibility, they increase nurses’ workload. Furthermore, many studies are concentrated in Western healthcare settings and involve novice teleconsultation providers, with inadequate representation of patients with rare diseases and nurses in low-resource settings (33).

In addition, nurse-led remote digital services for chronic disease management are delivered within nursing practice and mainly cover remote health monitoring, virtual health education and chronic self-management support, while existing relevant studies adopt highly diverse intervention forms, digital modalities and implementation arrangements that result in inconsistent intervention delivery and poor comparability of health outcomes (7).

In terms of innovative practice, nurse-led digital care models are evolving toward greater specialization and refinement. For instance, in the U. S.-based ELICIT study, systematically trained nurses delivered 12 months of digital palliative care via virtual video/audio platforms to community-dwelling older adults with chronic conditions and varying levels of cognitive impairment. The intervention covered four core components: symptom assessment, psychosocial support, communication of care goals, advance care planning, and care coordination, expanding nurses’ role in digital palliative care (36).

### 4.2 Multidimensional collaborative care models: from single provider to cross-setting integration

Multidimensional collaborative community-based care models overcome the limitations of standalone single-service delivery by establishing linkage between communities and formal medical institutions, enabling multidisciplinary collaboration among community health workers, clinical professionals and peer educators, and incorporating digital tools such as mHealth applications, remote service platforms and cloud-based data systems; such models broaden diabetes care coverage, facilitate health information integration, and constitute an essential scalable strategy for standardized type 2 diabetes management (2). The hospital–community hybrid model is led by community health workers and supported by digital tools, achieving seamless integration between hospitals and communities. In type 2 diabetes management, the digital–community hybrid intervention led by community health workers — using mHealth apps, SMS reminders, and telehealth — delivers diabetes self-management education and lifestyle interventions, significantly improving glycemic control and self-efficacy. Models integrated into existing health systems demonstrate higher sustainability (2). A narrative review conducted in Italy proposed a six-stage transitional care model led by case management nurses, including early screening, proactive planning, initiation of community services, caregiver education, remote follow-up, and outcome evaluation. Combined with predictive assessment tools (such as the BRASS score) and remote monitoring, this model reduces 30-day readmission rates, decreases unnecessary hospital days, and enhances care continuity and satisfaction among patients with chronic diseases (37).

The home-school / hospital-school collaborative model targets children and adolescents with chronic diseases by integrating family, school, and healthcare resources, enabling coordinated management via digital tools. In a U.S. cluster-randomized pilot trial, the SPACE collaborative care model for children aged 5–12 years with type 1 diabetes involved monthly 30-min online meetings (4 sessions total) among parents, school nurses, and diabetes educators to co-develop shared care plans. The recruitment rate reached 65%, and 93% of participants used continuous glucose monitoring systems, providing a feasible approach for chronic disease management in pediatric populations (38). In another U. S. technology-mediated training program, school nurses received fully online and blended modular training on common pediatric chronic diseases (e.g., diabetes, asthma) and on-campus emergency response via digital tools including learning management systems, video conferencing, virtual workshops, and the ECHO telehealth model, which significantly improved their clinical competencies (39).

Multidisciplinary collaborative models integrate expertise from pharmacists, dietitians, rehabilitation therapists, psychotherapists, and other specialists, enabling interprofessional coordinated interventions via digital tools. For example, in the U.S.-based EXTEND Plus study, nurse-led self-management support and pharmacist-coordinated medication management were added to mobile monitoring. The digital toolkit included glucose meters, blood pressure monitors, weighing scales, and activity trackers integrated with electronic health records. For patients with persistently poorly controlled type 2 diabetes complicated by hypertension, this approach is expected to improve hemoglobin A1c, blood pressure, and related clinical outcomes (40).

In terms of innovation, digital co-creation platforms have emerged as critical enablers for multidimensional collaborative care. In a randomized controlled trial conducted in China, a digital co-creation platform (CC-CoI) was established based on the Community of Inquiry framework. Delivered via WeChat and a pulmonary rehabilitation official WeChat account, the platform provided a 12-week nursing intervention for adults aged 65 years and older with stable chronic obstructive pulmonary disease (COPD), incorporating modules such as virtual avatars, visual learning, co-created comics, and virtual scenario training. Patients in the intervention group showed significantly better patient activation, adherence, self-efficacy, self-management, social support, and disease literacy, as well as greater relief from dyspnea (41).

Within the European iCARE4CVD initiative, the PRIC-CVD study engaged patient groups in core tasks and activities, continuously integrating patient perspectives into the development of digital tools. This work included establishing patient-reported outcome and experience measures to refine cardiovascular disease care pathways, exemplifying collaborative innovation centered on patient participation (42).

Multidimensional collaborative care models face challenges including inadequate coordination mechanisms, difficult resource integration, and poor interoperability of digital tools. Poor information-sharing mechanisms between hospitals and communities, coupled with limited interoperability of electronic health records, lead to fragmented care information (37). Within home–school collaboration models, school nurses are constrained by limited time and workload capacity; student recruitment also heavily relies on screening and referral by school nurses (38). Furthermore, existing digital precision hypertension management studies are geographically concentrated in limited regions, and their generalizability is constrained by regional disparities and socioeconomic inequities; in multidisciplinary research and practice, digital intervention tools remain fragmented and lack a unified integrated platform, hindering real-time cross-professional exchange of clinical, behavioral, and physiological data and undermining interdisciplinary coordination efficiency (43). In Sub-Saharan Africa, the delivery of non-communicable disease care relies on adequate healthcare workforce and medical resources, and shortages of staff and supplies hinder the effective provision of comprehensive diagnosis, treatment and follow-up health services in resource-limited regions (3).

### 4.3 Caregiver-enabled digital care models: from patient care to caregiver support

Informal caregivers of patients with chronic conditions (e.g., family members, relatives, and friends) undertake extensive care work including daily living assistance, disease management, and healthcare coordination. They face multiple caregiving burdens such as physical and psychological stress, social isolation, and fragmented health information (44). Caregiver-enabled digital care models provide tailored support to caregivers through digital means including informational resources, management tools, and psychological counseling, helping to alleviate caregiving strain and enhance caregiving competence.

#### 4.3.1 Core application dimensions of digital tools for caregiver empowerment

Digital support for informal caregivers spans multiple domains, including caregiving education, psychological support, and assisted symptom monitoring. In heart failure care, a U.S.-based randomized controlled trial equipped 250 informal caregivers of patients with chronic heart failure with Samsung tablets preloaded with accredited heart failure and caregiving-related websites; half of them received 10 synchronous virtual ViCCY health coaching sessions over 6 months, and at 6 months, the intervention group achieved statistically meaningful improvements in self-care maintenance and clinically significant reductions in perceived stress, while the between-group reduction in self-care neglect lost statistical significance after multiple comparison adjustment (45). In end-stage renal disease care, a pilot randomized controlled trial in Singapore supplied 30 primary caregivers of patients with end-stage renal disease with Oculus Quest 3 VR headsets and the TRIPP mindfulness application. Participants completed 10–15 min of daily VR-guided mindfulness training at home for 6 weeks, with the aim of reducing caregiving burden, stress, anxiety, and depression (46).

#### 4.3.2 Evidence-based outcomes of caregiver-empowering digital care models

Caregivers expect mobile health applications to satisfy their diverse needs covering care-related information, care support, care coordination and self-care, and they prefer apps featuring easy operation, electronic health record integration, real-time provider feedback and task reminders (44). Meta-analysis verified VR caregiver training including Dementia Live significantly improves caregivers’ overall caregiving competence, with a pooled effect size of 0.362 (95% CI: 0.181–0.543, *p* < 0.001), and Dementia Live separately yields statistically meaningful gains in caregiving competence (effect size = 0.322, 95% CI: 0.046–0.597, *p* = 0.022) (47). Virtual health coaching improves self-care maintenance and lessens perceived stress with both statistically and clinically meaningful effects, while producing statistically significant but clinically insignificant mental health gains for chronic heart failure caregivers (45).

#### 4.3.3 Existing challenges in caregiver-empowering digital care models

Digital care models for caregiver empowerment suffer from several limitations, including the neglect of caregivers’ digital literacy, incomplete support content, and lack of targeted interventions. Most digital tools fail to account for caregivers’ digital literacy levels; older and low-educated caregivers struggle to use digital health tools effectively due to inadequate digital health literacy. Existing interventions focus primarily on caregiving knowledge and skills training, with insufficient attention to caregivers’ needs for psychological, social, and financial support. Consequently, many caregivers experience unresolved anxiety, depression, and other mental health issues resulting from prolonged caregiving. Digital interventions targeting caregivers of patients with rare diseases or multimorbidity remain extremely scarce, leaving unique caregiving challenges unaddressed. Furthermore, collaborative interventions for caregivers and patients are inadequate. Most programs target either the caregiver or the patient individually, without developing integrated patient–caregiver dyadic digital intervention designs, which limits the overall effectiveness of care delivery.

#### 4.3.4 Innovative practices in caregiver-empowering digital care models

In terms of innovative practice, digital interventions for caregiver empowerment are evolving toward personalization and dyadic coordination. For example, the iCC-SCD digital cognitive behavioral therapy (CBT) program, conducted in the United States, provided a smartphone app plus website for adolescents aged 12–18 years with sickle cell disease and pain, along with a dedicated parent portal. Parental psychological distress significantly moderated the intervention effect of digital CBT on pain intensity, with greater analgesic benefits observed in families where caregivers reported higher levels of psychological distress (48). The prototype V-CCC digital resource was co-developed in Vietnam via a two-stage framework to provide informational, psychosocial and caregiving support for cancer caregivers, and both caregivers and healthcare professionals support the development of this web-based intervention (49). The three major care models-nurse-led, multidimensional collaborative, and caregiver-empowered-have reshaped the service system of digital chronic care. However, the suitability of each model remains influenced by population characteristics and disease types. The following section focuses on differences in applicability across diverse populations and conditions, and identifies current research gaps in digital care.

## 5 Population and disease adaptability of digital chronic disease care: the necessity of targeted interventions and research gaps

The effectiveness of digital interventions for chronic disease management is highly dependent on the adaptability to population characteristics and disease types. Significant differences exist in digital literacy and health needs among populations of different ages, ethnicities, and socioeconomic statuses. Meanwhile, the pathological characteristics and management priorities of various chronic diseases also differ substantially. Most existing studies adopt a one-size-fits-all standardized intervention model without targeted design, resulting in inconsistent intervention outcomes and numerous research gaps.

### 5.1 Analysis of digital care adaptability across different populations

Differences in the adaptability of digital chronic disease care are mainly reflected in dimensions such as digital literacy, cultural background, age-related physiological characteristics, disease complexity, and regional medical resources. Due to distinct core characteristics, older adults, ethnically diverse or immigrant populations, children and adolescents, individuals with multimorbidity, and populations in low- and middle-income countries exhibit significant heterogeneity in digital care barriers and adaptive needs. A detailed analysis is presented below.

#### 5.1.1 Older patients with chronic diseases: deficiencies in digital literacy and inadequate adaptive design

Older adults with cardiovascular diseases are a high-risk patient group, and their commonly insufficient digital literacy becomes a key barrier to adopting digital cardiovascular care technologies (12). A systematic review of 56 studies across 24 countries demonstrated that only 2 interventions targeting digital literacy training in older patients with cardiovascular diseases simultaneously improved technology utilization and health outcomes. Most studies merely confirmed the necessity of such training without demonstrating clear clinical benefits (12). Older patients’ advanced age limits immersive experience of VR equipment during chronic leg wound care, and a small number of them develop mild discomforts including eye strain, blurred vision and headache after VR application (26).

User-friendly design boosts both recruitment and retention of older adults with cardiovascular disease in digital care interventions, while printed manuals and on-demand technical helpline support effectively increase their study recruitment (12). In a randomized controlled trial conducted in South Korea, a digital health coaching program for community-dwelling older adults with multimorbidity, combining mobile applications, telephone guidance, and in-person education, significantly reduced health distress, alleviated depressive symptoms, and improved application-related digital health literacy (50).

However, most existing mHealth apps developed for family caregivers of patients with chronic conditions only deliver basic practical functions and fail to satisfy caregivers’ core needs amid complex care scenarios. Few available apps incorporate dedicated care coordination and care planning functions, including those targeting cancer and dementia—the two most-studied disease categories in existing research—thus leaving caregivers without effective digital tools to support systematic care management (44).

Excessive manual operations and poor integration with older adults’ daily routines reduce multimorbid older adults’ ongoing usage of digital health technologies. Although the digital platform was developed via user-centered participatory design processes, differences in device operation complexity and routine compatibility lead to three distinct user engagement clusters. Older adults with low engagement exhibited unstable physiological indicators and inferior multimorbidity self-management outcomes compared with highly engaged counterparts (51).

Digital health coaching interventions for community-dwelling older adults with multimorbidity have incorporated disease monitoring, medication management, psychological support, and limited social support functions. Nevertheless, such interventions lack dedicated modules addressing the needs associated with disability care; while the program yielded significant improvements in psychological indicators (distress, depression) and digital health literacy, it has not demonstrated significant group-by-time interactive improvements in core physical self-management behaviors, medication adherence and health-related quality of life (50).

During the COVID-19 pandemic, patients with noncommunicable diseases in sub-Saharan Africa faced substantial barriers to accessing healthcare services, closely linked to socioeconomic factors such as weak medical infrastructure and resource shortages in the region. Patients in rural areas encountered even greater challenges due to travel restrictions and uneven distribution of healthcare resources (3). While digital health technologies are regarded as a potential mitigating strategy, they have not yet been widely deployed to address these core access barriers.

#### 5.1.2 Racially/ethnically diverse and immigrant populations: insufficient cultural adaptation and trust building

Racially and ethnically diverse populations as well as immigrant communities face unique digital health barriers due to cultural and linguistic differences, low socioeconomic status, and insufficient trust. A review synthesizing 38 studies identified socioeconomic constraints, limited digital literacy, cultural and linguistic mismatches, and concerns regarding trust and privacy as the primary factors impeding digital health adoption among these groups (9). For instance, language barriers prevent many immigrants from comprehending health information presented in digital tools (52), while some ethnic populations are reluctant to share personal health data owing to privacy concerns about digital platforms.

Cultural tailoring and community engagement represent effective strategies to improve acceptance of digital care among these populations. As a culturally adaptive intervention, Digital Storytelling (DST) has shown promising potential among immigrant and refugee groups through culturally and linguistically tailored workshops, videos, and follow-up messaging. It can enhance health literacy, self-management confidence, and behavioral intentions related to type 2 diabetes, while significantly increasing HPV vaccination willingness among Asian immigrant mothers; in some contexts, it also promotes psychological well-being and social integration (52). Interventions led by community health workers paired with culturally tailored app development can effectively improve the acceptance and utilization of digital health applications among racially diverse populations per evidence from the reviewed studies and practical case examples (9).

Although existing culturally adapted DST interventions for immigrants and refugees commonly adopt language adjustment and culture-specific health storylines via community participatory design, relevant empirical studies have methodological limitations that hinder systematic verification of the full depth of their cultural adaptation effects (52).

Critical reflections indicate that digital health equity for racially diverse populations remains inadequately addressed. Most studies are dominated by White samples with low representation of racial and ethnic minorities, resulting in a lack of population-specific evidence regarding the effectiveness and safety of digital tools (43). Furthermore, trust-building mechanisms are underdeveloped: some patient groups may experience a crisis of confidence in digital health tools and healthcare systems due to historical experiences of medical injustice, which undermines intervention outcomes.

#### 5.1.3 Pediatric and adolescent patients with chronic diseases: strengths and limitations of home-school collaboration and gamified interventions

Notably, all pediatric and adolescent studies discussed in this section were excluded from the formal evidence synthesis of this review (per pre-specified eligibility criteria) and are presented here exclusively as comparative context to illustrate age-specific differences in digital care design and population adaptability. Digital collaborative care for children aged 5–12 years with type 1 diabetes centers on disease management and optimizes outcomes through virtual home-school-clinical collaboration. This digital integrated intervention linking schools, families, and healthcare teams represents an important innovative direction in chronic care for this population (38).

In a cluster randomized controlled pilot study conducted in the United States, the SPACE collaborative care model for children aged 5–12 years with type 1 diabetes involved monthly online meetings among parents, school nurses, and diabetes educators to co-develop shared care plans. The recruitment rate reached 65%, and 93% of participants used continuous glucose monitoring systems, providing a feasible model for chronic disease management in children and adolescents (38). A systematic review and meta-analysis demonstrated that telemedicine significantly reduced HbA1c by 0.22% and improved non-adolescent-specific quality of life in pediatric and adolescent patients with type 1 diabetes (53).

WeChat mini-program based digital gamified pursed-lip breathing (PLB) training, developed under the Behavior Change Wheel (BCW) framework, employs core modules including rankings, daily tasks, and social communities to enhance motivation and adherence among COPD patients during home-based rehabilitation. The safety, feasibility, and acceptability of this intervention are currently being evaluated in a pre-post feasibility study (20).

In a U.S.-based randomized controlled trial, Migraine Manager — a digital behavioral self-management tool for adolescent migraine — integrates cognitive behavioral therapy (CBT), problem-solving training, and health habit promotion. Preliminary pilot findings indicate that this tool significantly reduces headache days and improves quality of life (54).

Several limitations exist in digital health intervention research for chronic diseases in children and adolescents. For instance, most studies employ small sample sizes and show uneven geographic distribution: a disproportionate number originate from high-income countries, with far fewer conducted in low- and middle-income settings.

User engagement in digital health interventions for adolescents with chronic conditions declines noticeably over time. Many participants gradually reduce or discontinue use due to time costs associated with video uploads, inconsistent mobile phone access, and limited leisure time (55). Furthermore, excessive digital device use may contribute to vision deterioration and internet addiction. In addition, children and adolescents generally lack awareness of privacy protection, posing potential risks to data security.

#### 5.1.4 Populations with multimorbidity: the contradiction between homogeneous interventions and individualized needs

Patients with multimorbidity (defined as having two or more concurrent chronic conditions) present highly complex clinical profiles, which increase the difficulty of treatment and health management. This population is also at elevated risk of functional decline, disability, and premature mortality, representing a key focus of chronic disease management in global public health (56). Most existing digital health interventions for chronic diseases deliver generalized content with obvious homogeneous characteristics. They inadequately account for individual variations among patients with multimorbidity and lack personalized, precision care strategies tailored to specific patient characteristics (30). A systematic review including 13 studies demonstrated that nurse-led interventions can improve care quality, medication adherence, and self-efficacy in patients with multimorbidity (35). However, only 2 of these studies employed mHealth and other digital tools, and their intervention formats did not differ significantly from those designed for single chronic diseases.

Personalization and integration represent innovative directions in digital care for populations with multimorbidity. A three-arm parallel randomized controlled trial conducted in China utilized the AI-HEALS system based on traditional Chinese medicine constitution identification to deliver tailored health education for patients with multimorbidity (hypertension, type 2 diabetes, and hyperlipidemia), aiming to evaluate its effects on improving systolic blood pressure, blood glucose, lipid profiles, and other clinical indicators (30). A proof-of-concept study in Ireland identified three distinct engagement profiles in older adults with multimorbidity receiving digital care: low engagement, high engagement, and typical engagement. Device uniformity, number of operational steps, and alignment with daily routines were identified as key influencing factors, with the high-engagement group demonstrating more stable health outcomes (51).

Digital care for patients with multimorbidity faces multiple challenges. First, standardized and unified assessment methods are lacking. Among the 54 existing assessment tools for multimorbidity, four categories are recognized: disease count and equal-weight severity assessment, physical and mental health status evaluation, medication use assessment, and natural language processing–based systems. Although disease count or severity rating tools are widely applied, overall deficiencies remain in terms of disease coverage breadth, assessment depth, and data reliability, and no universally applicable unified assessment system has been established (56).

Second, most current digital interventions only provide generalized health education content, without sufficiently integrating individual Traditional Chinese Medicine (TCM) constitution characteristics and the unique needs of multimorbidity, making it difficult to achieve precise and root-cause-oriented individualized regulation (30).

Third, multiple obstacles hinder the integration between clinical practice and digital tools. A prominent challenge stems from fragmented medical data silos and heterogeneous data formats; poor interoperability between digital applications and clinical systems including electronic health records blocks seamless transmission of multi-source health data and impedes system integration (10).

#### 5.1.5 Populations in low- and middle-income countries: insufficient access and lack of locally adapted design

Demand for infectious disease control remains the top global health focus in LMICs, while chronic disease management is also an important emerging focus; research applying data science and AI to LMIC chronic diseases has been listed among expert-endorsed global research priorities. Global research prioritizes culturally tailored digital health and AI solutions for underserved populations across infectious and chronic disease care, and the study notes structural limitations including weak infrastructure and resource shortages create practical challenges for scaling up AI interventions such as chronic disease programs in LMICs, alongside unaddressed gaps in cultural adaptation (4).

In sub-Saharan Africa, chronic disease diagnosis and care services were severely disrupted during the COVID-19 pandemic (3). Although digital health communication and telemedicine emerged as mitigating strategies, their large-scale implementation was hindered by equipment shortages, poor internet coverage, and low digital literacy. A scoping review covering Latin America and the Caribbean indicated that telemedicine is feasible and well-received by patients in diabetes and hypertension management, yet lacks robust evidence of effectiveness, with limited rural data and acute resource shortages (57).

Locally adapted design is critical to improving the effectiveness of digital care in low- and middle-income countries (LMICs). Experts in LMICs prioritize research related to infectious disease control and diagnostic equity, whereas those in high-income countries (HICs) focus more on infrastructure optimization and climate-related health analyses, revealing distinct geographic differences in research priorities. Core needs in LMICs center on practical domains such as infectious disease prevention and healthcare access, while HICs emphasize system refinement and long-term health risk prediction (4). A relevant scoping review from India identified implementation challenges including limited internet access, technological gaps, linguistic and cultural disparities, and poor adherence to digital training programs among healthcare workers, highlighting the urgent need for locally tailored digital health education tools (58).

Digital care in low- and middle-income countries is often characterized by technology transfer rather than localized innovation. First, access to digital health tools remains severely limited. For example, although telemedicine approaches such as telephone and online consultations have been explored as mitigation strategies for noncommunicable disease (NCD) care in some regions, many patients cannot participate due to lack of reliable contact channels or requirements for in-person clinical evaluation (3). Second, barriers such as poor internet connectivity and limited digital literacy among healthcare workers hinder their participation in digital NCD training, and most existing Indian digital NCD training programs focus predominantly on clinical knowledge of chronic diseases with scarce published research regarding dedicated modules addressing practitioners’ practical digital learning hurdles (58). Third, high-income country experts and LMIC experts differ noticeably in the ranking order and core focus of prioritized AI and data science global health research topics (4). High-income country experts and LMIC experts show substantial divergence across all their ranked research priorities for AI and data science in global health.

### 5.2 Research characteristics of digital care for major chronic conditions

#### 5.2.1 Cardiovascular diseases: sufficient evidence for remote monitoring, with long-term efficacy yet to be confirmed

Cardiovascular diseases (including heart failure, hypertension, coronary heart disease, etc.) represent one of the most widely applied fields of digital care, with robust evidence supporting remote monitoring and nurse-led interventions. A systematic review demonstrated that remote monitoring enables early identification of worsening heart failure, and devices such as the CardioMEMS™ remote hemodynamic monitoring system significantly reduce hospitalization risk. However, some interventions including OptiVol failed to improve clinical outcomes (5).

Digital precision management of hypertension has emerged as a research hotspot. A scoping review of 46 studies found that 74% of included studies focused on personalized interventions, which mainly used mobile phones and blood pressure monitors to deliver tailored education, medication management and behavioral guidance; machine learning was utilized in a minority of all included studies, and most of these personalized interventions contributed to improved blood pressure control or medication adherence (43). A workshop report from the U. S. National Heart, Lung, and Blood Institute noted that AI can enhance cuffless blood pressure measurement, risk prediction, and personalized medication strategies for hypertension, yet faces challenges such as data bias and insufficient external validation (29).

Long-term efficacy and equity issues are prominent in digital care for cardiovascular diseases. Several trials including TIM-HF2 had follow-up within 1 year during active intervention, and extended follow-up of TIM-HF2 confirmed that the clinical benefits brought by its telemonitoring vanished 1 year after the intervention was withdrawn, leaving the long-term persistent benefits of such remote monitoring unproven (5). Research shows uneven geographic distribution: most relevant studies were conducted in North America and Europe, whereas only a small proportion came from Asia and South America and no eligible studies from Africa or Oceania were included in this review (43). In addition, 45% of studies on digital precision management for hypertension did not fully report racial and ethnic data, and only 7% included data on social determinants of health, reflecting insufficient attention to health equity (43).

#### 5.2.2 Diabetes mellitus (type 1 / type 2): diverse intervention forms with significant variations in population adaptability

Conversational agents (CAs) represent one of the core modalities in digital care interventions for diabetes, with diverse delivery platforms and functions. They are primarily deployed via mHealth apps and instant messaging platforms, with some utilizing humanoid social robots or voice-based interfaces. Their functions span disease education, medication management, glucose-related health coaching, and psychological support, providing multidimensional digital support for diabetes self-management (59). Within community-based models, blended digital-community interventions - centered on community health workers (CHWs) or peer support, supplemented by mHealth apps, SMS reminders, and other digital tools – effectively enhance self-management capabilities among patients with type 2 diabetes. Some of these interventions achieve clinically meaningful improvements in glycemic control (2).

Digital care outcomes for diabetes vary significantly across populations. Telemedicine interventions for children and adolescents with type 1 diabetes significantly reduce HbA1c and improve quality of life (53). Mobile video interventions for older adults with type 2 diabetes, grounded in the Information-Motivation-Behavioral Skills model, significantly enhance medication adherence and medication-related knowledge (17). Digital intelligent interventions for patients with diabetic foot (WeChat programs, mobile apps, electronic platforms) yielded significant self-management improvements in 17 of 18 RCTs, yet all trials had high risk of bias limiting definitive validation (60).

Digital diabetes care faces methodological flaws and insufficient clinical translation. Most studies exhibit low methodological quality: 12 studies were rated weak, and only 3 were rated moderate, with missing safety assessments and insufficient evidence on long-term effects (59). All included studies on digital interventions for diabetic foot were deemed to have a high risk of bias, with limitations such as methodological defects, high heterogeneity, and geographic bias (60). Clinical implementation of digital diabetes intervention tools such as conversational agents (CAs) faces notable practical constraints. Most CA interventions are developed as pilot prototypes and have not been designed to integrate into routine clinical workflows; only a small fraction of included studies embed clinical safety early-warning triggers and formal clinician escalation protocols, meaning existing evidence cannot sufficiently support translating CA-related intervention outcomes into uniform standardized clinical decision-making (59).

#### 5.2.3 Respiratory diseases (COPD / asthma): intelligent digital tools and VR rehabilitation as innovative directions

The core of digital health interventions for COPD lies in remote respiratory rehabilitation and disease self-management dominated by symptom monitoring and early exacerbation warning. Most digital tools supply rehabilitation guidance, symptom tracking and early warning, alongside auxiliary medication adherence support (61). Intelligent inhalers and matching smart spacers are innovative tools for asthma and COPD management to boost medication adherence, yet smart spacer-based inhalation education currently lacks sufficient large-sample long-term clinical evidence (62). Digital health interventions can significantly improve the quality of life and self-efficacy of COPD patients, reduce dyspnea, but have no significant improvement on the 6-min walk test, emergency visits, and hospitalization rates (61).

VR-assisted pulmonary rehabilitation has emerged as a research hotspot in the management of chronic obstructive pulmonary disease (COPD). A systematic review and meta-analysis including 16 randomized controlled trials (RCTs) demonstrated that VR-assisted pulmonary rehabilitation significantly improved patients’ forced expiratory volume in one second (FEV1), forced vital capacity (FVC), and 6-min walking distance, while alleviating dyspnea; the optimal intervention duration was 5–12 weeks, with higher patient adherence and acceptance (6). In randomized controlled trials conducted in China, a remote pulmonary rehabilitation intervention based on a digital co-creation platform significantly enhanced activation, adherence, and self-management ability among older patients with COPD (41).

However, digital care for respiratory diseases is plagued by technical homogeneity and insufficient population-specific adaptation. Standardization of VR-based interventions remains a prominent issue: substantial variations in equipment, intervention duration, and scenario design across studies hinder direct cross-study comparisons (63). Moreover, digital care interventions for older patients with COPD lack adequate design adaptability, failing to fully account for the characteristics of older populations. Low digital literacy and unfamiliarity with smart devices prevent some patients from effectively utilizing digital care tools such as remote monitoring and virtual consultations (64). Additionally, the integrative potential of inhalation drug delivery technology and digital tools has not been fully exploited. Issues including inadequate formulation stability and poor device compatibility further limit its clinical translation and application (62).

#### 5.2.4 Rare chronic diseases (sickle cell disease, Tourette syndrome): limited research and unverified feasibility

A certain empirical basis has been accumulated in the research on digital interventions for Chronic Tic Disorder (CTD) and Tourette Syndrome (TS). Three randomized controlled trials (RCTs) have verified the clinical effectiveness of online behavioral therapy and wearable neuromodulation devices; however, the research still has limitations, including a limited number of studies, as well as insufficient evidence on long-term cost-effectiveness and improvement of functional impairment (65).

A systematic review and health economic evaluation conducted in the United Kingdom showed that the Online Remote Behavioral Intervention for Tics (ORBIT) for chronic tic disorder and Tourette syndrome significantly reduced the total score of the Yale Global Tic Severity Scale (YGTSS) at 3 and 12 months, and the wrist-worn neuromodulation device Neupulse significantly reduced this score at 4 weeks. However, neither of them improved the tic impairment score, and their cost-effectiveness remains highly uncertain (65).

Digital care for sickle cell disease (SCD) primarily focuses on pain management and self-management. A multicenter randomized controlled trial conducted in the United States demonstrated that digitally delivered cognitive behavioral therapy (CBT) supported by health coaches significantly improved pain interference, depressive symptoms, and anxiety, as well as social functioning and emotion-related quality of life among adults with SCD. These benefits were comparable to those observed with a health coach–supported digital pain and SCD education intervention (19). Virtual reality-based home intervention program incorporating immersive distraction alongside multidisciplinary pain-management skills training is feasible and shows promising acceptability for adults with sickle cell disease experiencing chronic pain (66). Additionally, iCanCope with SCD (iCC-SCD), a digital cognitive behavioral self-management tool for adolescents with SCD, significantly reduced pain intensity and the number of pain days, and improved pain-related coping attempts. Notably, pain reduction was more pronounced among adolescents whose parents experienced psychological distress (48).

Digital care for rare chronic diseases faces dual challenges: large evidence gaps and insufficient resource investment. Existing studies are characterized by small sample sizes and limited evidence, with a lack of large-sample, multicenter randomized controlled trials (RCTs), inconsistent outcome measures, and a scarcity of long-term data (65). There is also a notable deficit in digital tools designed for family caregivers. Current tools mostly target single needs such as caregivers’ mental health and disease education, while dedicated interventions supporting care planning and coordination, multi-stakeholder communication, and integration with electronic health records (EHRs) remain underdeveloped. Moreover, relevant research is predominantly pilot-based or qualitative, lacking rigorous empirical support (44). Patients with sickle cell disease (SCD) exhibit substantial clinical heterogeneity and personalized requirements. Differences in cognitive function, daily schedules, and other factors may affect engagement with digital interventions among some patients. Although existing standardized home-based virtual reality (VR) intervention protocols can fulfill basic pain management needs, further personalized tailoring is required to improve their adaptability and fit (66).

#### 5.2.5 Chronic disease comorbidity / chronic pain: digital cognitive behavioral therapy (CBT) serves as the primary intervention, with family factors exerting a substantial impact

Digital care for chronic disease comorbidity in patients with inflammatory bowel disease (IBD) complicated with physical and psychological symptoms mainly adopts digital cognitive behavioral therapy (CBT) as the primary intervention form (67). A multicenter randomized controlled trial conducted in the United Kingdom showed that the IBD-BOOST digital cognitive behavioral self-management program combined with usual care could improve fecal incontinence and generic quality of life in patients with IBD, and patients who adhered to the intervention had a significant improvement in disease-specific quality of life (67). A mixed effectiveness-implementation randomized controlled trial carried out in the United States plans to verify the effect of digital CBT on pain management in patients with recurrent acute pancreatitis or chronic pancreatitis accompanied by chronic pain (18).

Family factors exert a significant influence on the effectiveness of digital care for chronic multimorbidity and chronic pain. A secondary analysis of a joint US-Canadian randomized controlled trial showed that only parental psychological distress significantly moderated the analgesic effect of digital cognitive behavioral therapy (CBT) among adolescents with sickle cell disease pain; the analgesic benefit of this intervention was stronger in families with higher levels of parental psychological distress (48). Insufficient caregiver support among patients with chronic multimorbidity can disrupt the maintenance of patients’ care routines, thereby leading to adverse health outcomes (44).

Digital care for chronic multimorbidity and chronic pain is plagued by issues of intervention homogeneity and heterogeneous outcomes. The digital CBT-based IBD-BOOST trial employed only loose enrolment criteria without refined stratified screening accounting for inter-individual discrepancies including concomitant IBS, varying pain severity and differing psychological profiles (depression and visceral anxiety); the study authors hypothesized that this broad, unrefined participant selection represented one plausible factor underlying the non-significant primary trial endpoints (67). One study demonstrated that the effects of digital CBT interventions in adolescents with sickle cell disease were moderated by family factors (48), resulting in substantial heterogeneity across studies and hindering the development of uniform intervention recommendations. Several investigations also reported low intervention adherence; for instance, in the IBD-BOOST trial, intention-to-treat analysis revealed no statistically significant between-group differences in the primary outcome, with benefits observed only among participants who adhered to the intervention (67).

Significant disparities exist in the suitability of digital care across different populations and diseases. Issues such as homogeneous interventions and the lack of culturally adapted, locally tailored designs have emerged as core bottlenecks. In light of the aforementioned research gaps and existing challenges, this paper will propose key directions and supportive strategies for the future development of digital care for chronic conditions.

## 6 Limitations of this review

While this review offers a systematic synthesis of the technical systems, care models and practical characteristics of digital care for chronic diseases, several scope- and methodology-related considerations should be noted when interpreting the findings.

First, the literature search is defined within a focused scope with notable methodological trade-offs. The search was conducted exclusively in the PubMed database and restricted to free full-text publications released between 2021 and 2026, to prioritize peer-reviewed studies with accessible full-text evidence for in-depth narrative synthesis. This scope setting carries two key limitations: (1) single-database selection bias, as relevant studies indexed only in Embase, CINAHL, Scopus or regional databases may have been omitted; (2) open-access selection bias, as subscription-only publications and gray literature were excluded from the formal evidence synthesis. In addition, the search terms were framed around nursing-focused digital interventions, so studies from public health, medical informatics or health economics perspectives may be underrepresented.

Second, there is a certain degree of heterogeneity across included studies. The incorporated literature covers multiple research designs, and the selected digital interventions vary in technical form, implementation setting, target population, intervention intensity and outcome measurement approaches. Given this heterogeneity across studies, this review adopted narrative synthesis rather than quantitative meta-analysis, so cross-study comparisons of intervention efficacy should be interpreted with appropriate caution.

Third, the inclusion and exclusion criteria define the applicable scope of the conclusions. This review centered on adult patients with chronic diseases, and excluded pediatric chronic disease populations, professional caregiver training research and pure technology development studies without clinical application scenarios. The five-year time frame was chosen to capture the latest advances in digital care, but it also means foundational studies with longer-term follow-up are not included in the formal evidence synthesis. Accordingly, the generalizability of findings to broader populations and longer-term practice contexts remains to be further verified.

Overall, these limitations are common in narrative reviews with a clear thematic focus, and do not undermine the core value of this review in sorting out the current research landscape and identifying key research gaps.

## 7 Future outlook of digital care for chronic conditions

In response to the above issues, we propose four core development directions centered on precision, localization, equity, and integration. Among them, precision serves as the core goal to resolve intervention homogeneity; localization constitutes the foundation for adapting to regional resources and achieving sustainable development; equity represents the core value pursuit to improve accessibility for all populations; and integration is the critical implementation pathway to break down barriers between data and scenarios. These four dimensions are mutually supportive and advance synergistically, while a sound policy and ethical guarantee system is required to consolidate the foundation for development. Although digital care for chronic conditions has formed a developmental landscape featuring diverse technological systems and multiple care models, it still faces prominent challenges, including insufficient high-quality evidence, a pronounced digital divide, difficulties in clinical integration, homogeneous intervention design, and a lack of localized innovation. Future development of chronic disease digital care should take precision, localization, equity, and integration as core directions, promote the deep integration of technological innovation with clinical needs, strengthen targeted interventions for vulnerable populations, conduct localized long-term studies, and improve policy and ethical safeguards. A fundamental shift from “technology-driven” to “evidence-driven and demand-driven” models should be realized, ultimately enhancing the overall efficiency of chronic disease management and health equity. This section focuses on the four core directions, proposes targeted development strategies and research priorities in light of existing research gaps and practical obstacles, and clarifies the policy and ethical guarantee systems that support the sustainable development of digital care.

### 7.1 Precision: targeting the cross-sectional needs of populations and diseases to achieve individualized digital care

Precision is the core pathway to address the current homogeneity and insufficient adaptability of digital interventions for chronic diseases. Its essence lies in establishing a closed-loop precision care system of “assessment-intervention-feedback” based on population characteristics and differences in disease pathology. In current studies, prominent issues—such as generalized interventions for populations with multiple comorbidities, a lack of targeted tools for rare diseases, and insufficient clinical translation of precision assessment technologies—have restricted the practical effectiveness of digital care. In the future, breakthroughs need to be made in three dimensions: tool development, technology optimization, and population focus.

#### 7.1.1 Develop population-disease cross-targeted intervention tools

To address the complex needs of patients with multiple comorbidities, it is necessary to strengthen the precise matching of “individual characteristics - disease combinations - intervention schemes”. Chinese researchers have designed a three-arm parallel randomized controlled trial, aiming to explore the AI-HEALS system based on TCM constitution identification, which provides customized health education interventions for patients with chronic multimorbidity complicated with hypertension, type 2 diabetes mellitus, and hyperlipidemia, in order to verify the application value of this model in improving patients’ clinical indicators (30). In the future, it is necessary to expand the sample size and follow-up period of such studies, include more types of chronic disease combinations, and integrate functions such as drug interaction early warning and multi-disease priority management to form an exclusive digital intervention framework for multiple comorbidities.

For rare chronic diseases, it is necessary to fill existing research gaps and develop disease-specific digital tools. British studies have shown that Online Remote Behavioral Intervention for Tics (ORBIT) and the wrist-worn neuromodulation device Neupulse can improve symptoms of chronic tic disorder and Tourette syndrome, but their long-term effects and cost-effectiveness remain unclear (65); A series of US-based studies on sickle cell disease (SCD), a rare hereditary hematologic disorder, corroborates this pattern. Lightweight digital cognitive behavioral therapy (CBT) interventions including the adolescent-oriented iCC-SCD program and the Facebook Messenger-delivered digital CBT platform, alongside home-based virtual reality (VR) devices, have proven effective in reducing the frequency of chronic pain episodes and improving patients’ pain coping capacities. Nevertheless, most relevant trials adopt small-to-moderate sample sizes with limited follow-up durations despite their multicenter design. Major research gaps remain regarding long-term clinical efficacy, health economic evidence, and digitalized supportive programs for caregivers. Future research should prioritize iterative optimization of tailored digital therapeutics targeting core painful manifestations and caregiver burden, coupled with successive multicenter small-scale clinical trials to accumulate robust clinical evidence (19, 48, 66). In the future, we should focus on the core symptom management and caregiver support of rare diseases, develop lightweight and highly adaptable digital tools, conduct multi-center small-sample clinical trials, and accumulate targeted evidence.

#### 7.1.2 Optimize precision assessment and intelligent intervention technologies

The in-depth application of AI and big data technologies is the key support for precision. A workshop report from the National Heart, Lung, and Blood Institute (NHLBI) indicates that AI can optimize cuffless blood pressure measurement for hypertension and improve the level of individualized medication, but there are issues of data bias and insufficient validation (29). In the future, it is necessary to strengthen the localized training of AI algorithms, incorporate data from special populations such as those with racial diversity and comorbidities, and improve the accuracy of risk prediction models; there are obvious shortcomings in the construction of big data-driven health profiles for non-communicable diseases (NCDs). It is necessary to strengthen the integration of attribute data in fields such as lifestyle and psychological status to make up for the current lack of data diversity. At the same time, it is necessary to specifically improve the compliance rate of the 3V (Volume, Velocity, Variety) standards and the proportion of external validation. Currently, these two indicators are only 17.78 and 30.34% respectively, and only 10.11% of studies meet both requirements, which is a key gap restricting the practicality and promotion of health profiles (28). In terms of precision intervention technologies, the Switzerland-developed AI wound assessment tool achieves high-precision wound segmentation and relatively reliable tissue classification, with mobile inference latency limited to only 0.3 s, which provides solid technical support for clinical precise intervention (31). In the future, efforts should be made to promote the in-depth integration of such technologies with clinical workflows, establish a closed-loop management of “data collection - risk early warning - intervention implementation - effect feedback”, and expand to more chronic disease complication scenarios such as diabetic foot and chronic ulcers.

### 7.2 Localization: adapting to regional resource characteristics to build a sustainable digital care ecosystem

Currently, digital care for chronic diseases is plagued by a problem where “technology transplantation” outweighs “localized innovation.” Prominent phenomena—such as insufficient accessibility of tools in low- and middle-income countries (LMICs) and the disconnection between technologies in high-income countries and local needs—have restricted the equity and sustainability of global chronic disease management. In the future, it is necessary to build a localized digital care system based on the economic level, medical resources, cultural characteristics, and disease spectrum of different regions.

#### 7.2.1 Develop low-cost digital tools adaptable to resource constraints

Given the current situation of resource scarcity in LMICs, it is necessary to break through the economic and technological barriers of commercial devices and develop low-cost digital tools with low digital literacy requirements. During the COVID-19 pandemic, chronic disease diagnosis and treatment services in sub-Saharan Africa suffered a severe disruption, which was specifically manifested by significant restrictions on outpatient visits, diagnostic surgeries, follow-up care, and laboratory testing services. Digital health communication and telemedicine have been gradually adopted as important strategies to alleviate this predicament, used for remote follow-up of chronic disease patients and sharing of diagnosis and treatment experience. However, their application scope is still limited due to factors such as some patients lacking effective contact information and some cases requiring on-site clinical assessment (3). Against a heavy non-communicable disease burden, India adopts digital education to strengthen medical practitioners’ ability to prevent, diagnose and manage prevalent chronic diseases like diabetes and hypertension. At present, online self-study, blended learning and MOOC have been rolled out for relevant training. Given India’s multilingual context and uneven digital infrastructure across urban and rural areas highlighted in the review, researchers suggest generating context-specific guidelines grounded in local empirical evidence and conducting rigorous domestic research to improve future digital NCD education in India (58).

#### 7.2.2 Conduct localized Long-term studies and model innovation

Differences in disease spectra and health needs across regions require the localized adaptation of digital care models. A global study on AI research priorities shows that experts in LMICs focus more on infectious disease control and diagnostic equity, while those in HICs prioritize infrastructure and climate-related analytics, and this difference highlights the need for regionally adaptable research agendas (4). In Latin America and the Caribbean, telemedicine is feasible in the management of diabetes and hypertension with high patient satisfaction, but it lacks effectiveness evidence and data from rural areas (57). In the future, it is necessary to conduct long-term cohort studies targeting this region to verify the long-term effects of telemedicine, and optimize the service radius in combination with the distribution of community medical resources.

For high-income countries, it is necessary to focus on the localized needs of special populations. Norway’s digital outpatient service (based on the Dignio Connected Care platform) provides flexible services for adult patients with long-term health needs such as cancer, interstitial lung disease, epilepsy, or complex pain through functions such as asynchronous communication, personalized task reminders, and collection of patient-reported outcomes (PROs). Patient subjective experiences show that this model can support self-management, improve medical convenience and a sense of security, and enhance psychological security through convenient contact with medical staff, which aligns with the core needs of patients with chronic diseases for independent care and flexible services (11). In the future, it can further integrate home care resources to form a hybrid model of “digital tools + home visit services”.

### 7.3 Equity: bridging the digital divide to improve accessibility for all populations

Health equity is the core value pursuit of digital care for chronic diseases. Currently, the digital divide has become an important factor exacerbating health inequalities, with prominent issues of insufficient accessibility of digital care among older adults, ethnically diverse groups, rural and low-income populations. In the future, efforts should be made from three dimensions—technological accessibility, capacity building, and cultural adaptation—to narrow the digital divide:

#### 7.3.1 Establish a hierarchical digital literacy training system

Insufficient digital literacy is the main obstacle for special populations to use digital care tools. Based on the findings of this randomized controlled trial, the existing DHCSMP-MCC program adopts a dual core training content consisting of digital tool operation and multimorbid chronic disease self-management, alongside multiple adaptive delivery modes including offline in-person practical training, individualized weekly telephone health coaching, and full-scale technical support covering remote online troubleshooting and on-site in-person assistance. This integrated model effectively improves older solo-living multimorbid adults’ proficiency in operating designated digital health tools (custom mobile APP and smart wearable monitoring bands) and significantly elevates their digital health literacy, especially the subdomain of mobile application usage (50). Lack of targeted specialized training and dedicated practice support guidelines ranks among the major obstacles impeding primary care nurses’ virtual care role enactment globally. Inadequate training undermines nurses’ practical performance in their three predominant virtual core duties: remote patient triage and assessment, chronic disease remote management, and cross-setting care coordination. Given most current virtual care practice resources are designed for general clinicians or physicians instead of nurses, formulating exclusive training programs and profession-specific practice standards for primary care nurses is indispensable to meet the practical needs of primary care virtual healthcare provision.

#### 7.3.2 Promote low-threshold technologies and culturally adaptive interventions

For racially diverse and immigrant populations, it is necessary to address the issues of cultural and linguistic mismatches and insufficient trust. Narrative reviews have shown that socioeconomic constraints, limited digital literacy, cultural and linguistic differences, and privacy concerns constitute the primary barriers preventing this group’s adoption of digital health applications (9). For future practice, DST should be culturally tailored and produced in multiple languages to reduce cultural and linguistic barriers and improve the comprehensibility of health information among immigrant and refugee populations (52); at the same time, build a trust bridge through interventions led by community health workers to enhance the acceptance and utilization rate of digital tools.

For rural and low-income populations, it is necessary to break through the barriers of equipment and the Internet. Empirical evidence from a small number of LMICs (China and Iran) confirms workplace wellness programs based on WeChat (multiple Chinese workplace settings) or WhatsApp (one single Iranian hospital nursing worksite) are feasible in their respective local settings (68). In the future, we can promote the service model of “equipment sharing + public network”, configure public digital devices in community health centers, and develop offline functional modules to reduce network dependence; for chronic diseases such as hypertension, we can promote a digital intervention model based on mobile applications. Mobile applications equipped with health data recording, medication reminder and professional health guidance functions can help adult and older patients with hypertension and diabetes strengthen disease self-management, boost medication adherence and improve disease-related healthy behaviors, so as to deliver effective digital technical support for the routine basic management of these two chronic diseases (69).

### 7.4 Integration: breaking down scenario and data barriers to build a collaborative care system

Currently, digital care for chronic diseases is faced with problems such as fragmented tools, poor collaboration mechanisms, and difficulties in clinical integration. Insufficient information sharing in multi-dimensional collaborative care and poor interoperability between digital tools and Electronic Health Records (EHR) have restricted care efficiency. In the future, it is necessary to break through the barriers of technology, models and data with an integrated thinking, and build an integrated digital care system of “hospital-community-family.”

#### 7.4.1 Build an integrated digital care platform

Break the fragmented status of tools, integrate functions such as monitoring, intervention, communication, and management, and achieve seamless connection across multiple scenarios. The Chronic Obstructive Pulmonary Disease (COPD) Digital Co-Creation Platform (CC-CoI) launched in China integrates the entire process of remote pulmonary rehabilitation services through modules such as virtual avatars, visual learning, and virtual scenario training, which has significantly improved patients’ activation level and compliance (41). We can further optimize the integrated digital platform for chronic disease management, realize the data integration of wearable monitoring devices, mobile applications and Electronic Health Records (EHR) relying on the existing technical architecture, and support the transmission of patient-generated health data to EHR and the formation of clinical alerts; at the same time, retain the collaborative intervention model of nurses, pharmacists and doctors, with nurses leading the implementation of interventions, pharmacists responsible for drug management, and doctors supervising treatment adjustments, to provide technical and professional support for complex chronic diseases such as refractory diabetes combined with hypertension (40).

#### 7.4.2 Improve the multi-subject collaborative care mechanism

Strengthen the core role of nurses in leading collaboration, and optimize the integrated model of multidisciplinary collaboration and caregiver empowerment. Nurse-led digital interventions have been proven to improve patients’ clinical indicators and self-management abilities (7). In the future, it is necessary to clarify the coordinating role of nurses in digital care and establish a hierarchical collaboration process of “nurses – specialists – community health workers”; for children and adolescents with type 1 diabetes, promote the home-school-medical collaborative model (“SPACE collaborative care”) of “parents – school nurses – specialist educators,” and jointly formulate care plans through online meetings (38).

At the same time, strengthen the collaborative intervention between caregivers and patients, and develop dual-end digital tools for “patients - caregivers.” Based on secondary analysis of a multicenter randomized controlled trial conducted across the United States and Canada, the dual-module iCC-SCD digital CBT intervention equipped with a dedicated smartphone app for adolescents and an independent website for caregivers yields superior reductions in pain intensity among adolescents with sickle cell disease whose caregivers present elevated baseline psychological distress, and pre-intervention parental psychological distress is confirmed as the sole statistically significant moderator affecting the intervention’s efficacy in lowering pain intensity (48). In the future, we should expand the application scenarios of such collaborative tools, cover chronic diseases such as dementia and cancer, integrate functions such as care knowledge training, psychological support, and collaborative monitoring, and reduce the care burden.

### 7.5 Policy and ethical safeguards: building a cornerstone for the sustainable development of digital care

Technological innovation and model optimization must rely on a sound policy and ethical system. Currently, digital care for chronic diseases faces ethical challenges such as data privacy and security, algorithmic bias, and lack of supervision, while also lacking targeted policy support and standard specifications. In the future, it is necessary to build a guarantee system from three dimensions: policy guidance, ethical norms, and quality supervision.

#### 7.5.1 Strengthen policy guidance and resource guarantee

Formulate special policies for chronic disease digital care, clarify technical application standards, clinical access procedures, and medical insurance payment mechanisms. Studies on primary non-communicable disease prevention and control policies at the provincial level in China’s Heilongjiang and Guangdong provinces indicate that underdeveloped health information systems are a prominent shortcoming, together with lingering shortages in health financing and workforce capacity (70). In the future, it is necessary to increase investment in digital care infrastructure, promote the construction of regional health data sharing platforms; include eligible digital care services into the scope of medical insurance payment, and encourage medical institutions to carry out digital care services.

#### 7.5.2 Standardize ethics and data security management

Establish ethical review standards for digital care to prevent algorithmic bias and data privacy risks. Studies on the application of AI in chronic disease self-management have shown that algorithms often have biases due to the lack of representativeness of training data (such as single-center, narrow-range cohorts) and the absence of external validation across populations and regions, which limits the scalability and generalizability of the tools (10). In the future, it is necessary to formulate ethical review standards for algorithmic fairness, requiring the inclusion of diverse population data for training and validation; regarding the privacy protection of digital health applications, it is necessary to establish transparent data privacy practices and deliver transparent communication about data handling rules, enhance user trust through strengthened data protection measures, and alleviate the privacy concerns of diverse ethnic groups regarding data leakage, abuse and surveillance (9).

#### 7.5.3 Establish a quality supervision and effect evaluation system

Formulate clinical verification standards and quality rating systems for digital care tools to prevent low-quality tools from entering clinical practice. Most existing studies regarding conversational agent (CA) applications in diabetes care are of poor methodological quality, and only a minority of relevant research has incorporated formal safety assessment (59). In the future, it is necessary to establish third-party quality assessment institutions to conduct ratings from dimensions such as evidence-based evidence, user experience, and data security; For the application of big data-driven health profiles in non-communicable diseases (NCDs) management, it is necessary to conduct more rigorous empirical research, strengthen external validation and multi-dimensional data integration aligned with the big data 3 V framework, so as to lay a foundation for quality optimization and practical implementation of health profiles targeting disease diagnosis, risk stratification and personalized intervention (28).

## 8 Conclusion

The global epidemic of chronic diseases has become a severe public health challenge, and the inherent drawbacks of the traditional care model, such as uneven allocation of medical resources, insufficient care continuity, and poor patient self-management capacity, have become increasingly prominent. Driven by the rapid evolution of digital technologies and further catalyzed by the COVID-19 pandemic, digital nursing has emerged as a pivotal approach to the transformation of chronic disease management, ushering in a new stage of integrated development of technology and clinical care. Based on relevant studies published in recent years, this review takes the logical chain of “evidence-based evidence – care model – population adaptation – practical implementation” as the core, systematically combs the technical system and model innovation characteristics of digital nursing for chronic diseases, deeply analyzes the adaptation differences among different populations and disease types, and conducts an in-depth critical reflection on the current dilemmas in research and practice. On this basis, it proposes the core development directions of “precision, localization, equity, and integration,” and constructs a comprehensive development framework for digital nursing for chronic diseases with policy and ethical guarantee as the cornerstone.

This study finds that digital nursing for chronic diseases has formed a multi-dimensional and comprehensive development pattern in its development, and has achieved remarkable progress in both technical application and care model innovation. In terms of the technical system, four core systems have taken shape, including intelligent perception and remote monitoring, digital behavioral intervention and cognitive therapy, immersive technologies such as VR/AR/MR, and AI and big data technology. These systems have realized the whole-process coverage of chronic disease management from real-time health data collection and early risk warning to personalized behavioral intervention and intelligent clinical decision support, and have been verified to have positive effects in improving patients’ clinical indicators, enhancing self-management ability and reducing the consumption of medical resources. In terms of care models, the traditional single-subject care model dominated by medical staff has been gradually reconstructed, and a diversified care system featuring nurse-led practice, multi-dimensional collaboration and caregiver empowerment has been formed. Nurse-led digital nursing has played a core role in care coordination and remote management; multi-dimensional collaborative care has broken the boundaries of medical scenarios and professional disciplines, effectively improving the coverage and information integration of chronic disease care; caregiver-empowered digital nursing has filled the gap in the support system for informal caregivers, forming a joint force for comprehensive management of chronic diseases. Together, these innovations have injected new vitality into the long-term management of chronic diseases and provided a feasible path for solving the pain points of the traditional care model.

However, the current development of digital nursing for chronic diseases is still in a stage of exploration and improvement, and there are still prominent problems and challenges that restrict its large-scale clinical transformation and sustainable development. At the technical level, the disconnection between data collection and clinical application is prominent, most digital interventions have obvious short-term effect bias, and the long-term efficacy and stability need to be further verified; AI and big data technologies are plagued by algorithm bias, insufficient external verification and prominent data privacy and security issues, and the clinical transformation efficiency of most technical tools is low. At the model level, nurse-led digital nursing is restricted by the uneven digital health literacy of nursing staff and the lack of standardized training systems; multi-dimensional collaborative care faces practical obstacles such as poor coordination mechanisms, low interoperability of digital tools and insufficient resource support; caregiver empowerment lacks personalized intervention design and multi-subject collaborative support, making it difficult to meet the diverse needs of caregivers. At the level of population and disease adaptation, the digital divide among older adults, ethnic diverse groups, and populations in low- and middle-income countries is prominent, and the “one-size-fits-all” standardized intervention model is difficult to adapt to the personalized needs of patients with multiple comorbidities and rare chronic diseases; the research on digital nursing for special diseases and groups is relatively scarce, and the high-quality evidence system is not yet perfect. In addition, the overall quality of evidence-based research on digital nursing for chronic diseases needs to be improved, the phenomenon of “technology transplantation” is obvious in practical application, and the localization innovation capacity is insufficient, which further restricts its popularization and application in different economic and medical resource contexts.

To address the above problems, the future development of digital nursing for chronic diseases must realize the fundamental transformation from “technology-driven” to “evidence-driven and demand-driven,” and take precision, localization, equity and integration as the core development directions, with the four dimensions supporting and promoting each other. Precision is the core goal to solve the problem of intervention homogenization, which requires the development of population-disease cross-targeted intervention tools, the optimization of precise assessment and intelligent intervention technologies, and the construction of a closed-loop precise care system of “assessment - intervention - feedback” to meet the personalized management needs of different patients. Localization is the basic premise for realizing sustainable development, which needs to adapt to the regional differences in economic level, medical resources, cultural characteristics and disease spectrum, develop low-cost and high-adaptability digital tools, and carry out long-cycle localized research and model innovation to avoid blind technical application. Equity is the core value pursuit of digital nursing development, which needs to focus on bridging the digital divide, construct a hierarchical digital health literacy training system, promote low-threshold digital technologies and culturally adapted interventions, and improve the accessibility of digital nursing for all populations, especially vulnerable groups. Integration is the key implementation path to improve the efficiency of chronic disease management, which needs to build an integrated digital nursing platform, break the fragmentation of tools and data islands, improve the multi-subject collaborative care mechanism, and form an integrated digital care system of “hospital - community - family” to realize the seamless connection of chronic disease care in different scenarios.

In addition, the healthy and sustainable development of digital nursing for chronic diseases cannot be separated from a sound policy and ethical guarantee system. It is necessary to strengthen policy guidance and resource support, formulate special policies and technical application standards for digital nursing for chronic diseases, improve the medical insurance payment mechanism for digital nursing services, and increase investment in digital nursing infrastructure construction to lay a solid material foundation for its development. It is essential to standardize ethical review and data security management, establish a sound algorithm fairness evaluation system, improve the data privacy protection mechanism, and effectively guard against ethical risks such as algorithm bias and data abuse. It is also required to build a scientific quality supervision and effect evaluation system, formulate clinical verification standards and quality rating systems for digital nursing tools, and set up third-party evaluation institutions to ensure the safety, effectiveness and standardization of the clinical application of digital nursing technologies.

In summary, digital nursing is an inevitable trend in the development of chronic disease management in the digital age, with broad application prospects and huge development potential. Facing the current development dilemmas, the global medical and health community needs to adhere to the people-centered concept, take the deep integration of technological innovation and clinical needs as the starting point, strengthen cross-disciplinary, cross-regional and multi-institutional cooperation and research, and focus on solving the key problems of insufficient evidence quality, prominent digital divide, difficult clinical integration and insufficient localization innovation. Through the joint efforts of technological research and development, model optimization, policy support and ethical constraint, digital nursing for chronic diseases will be further improved in terms of precision, localization, equity and integration, and its role in the global chronic disease prevention and control system will be fully exerted, so as to continuously improve the overall efficiency of chronic disease management and the level of health equity, and contribute to the realization of the goal of universal health coverage and the improvement of the health quality and life expectancy of chronic disease patients worldwide.

## References

[1] Global Burden of Disease 2019 Cancer Collaboration Kocarnik JM Compton K Dean FE Fu W Gaw BL . Cancer incidence, mortality, years of life lost, years lived with disability, and disability-adjusted life years for 29 cancer groups from 2010 to 2019: a systematic analysis for the global burden of disease study 2019. JAMA Oncol. (2019) 8:420. doi: 10.1001/jamaoncol.2021.6987, 34967848 PMC8719276

[2] Esht V Verma M Malik S Slimani MAM Kaur G Tripathy JP . Community-based models for type 2 diabetes care: A review of effectiveness, implementation, and health system integration. Adv Ther. (2026) 43:982–1003. doi: 10.1007/s12325-025-03462-7, 41498880 PMC12999817

[3] Kidanemariam YT Abebe F Girma E Addisse A Birhanu Z Hassen K . Impact of COVID-19 pandemic on non-communicable diseases care and service deliveries in sub-Saharan Africa: A systematic review. BMC Health Serv Res. (2025) 25:1075. doi: 10.1186/s12913-025-13270-y, 40804722 PMC12351779

[4] Song P Jiang D Zhou J Zhu Y Manaf RA Bojude DA . Research priorities for data science and artificial intelligence in Global Health: an international consensus exercise. Lancet Glob Health. (2026) 14:e455–65. doi: 10.1016/S2214-109X(25)00473-5, 41713447

[5] Alvarez P Sianis A Brown J Ali A Briasoulis A. Chronic disease Management in Heart Failure: focus on telemedicine and remote monitoring. Rev Cardiovasc Med. (2021) 22:403–13. doi: 10.31083/j.rcm2202046, 34258907

[6] Chen Y Zhang Y Long X Tu H Chen J. Effectiveness of virtual reality–complemented pulmonary rehabilitation on lung function, exercise capacity, dyspnea, and health status in chronic obstructive pulmonary disease: systematic review and meta-analysis. J Med Internet Res. (2025) 27:e64742. doi: 10.2196/64742, 40193185 PMC12012404

[7] Kilfoy A Chu C Krisnagopal A Mcatee E Baek S Zworth M . Nurse-led remote digital support for adults with chronic conditions: A systematic synthesis without Meta-analysis. J Clin Nurs. (2025) 34:715–36. doi: 10.1111/jocn.17226, 38894583 PMC11808464

[8] Towett G Snead RS Marczika J Ambalavanan R Kairichi MM Malioukis A. Bridging the gap in digital health: A framework for leveraging digital health Technologies in Cardiovascular Diseases, hypertension, and diabetes—a narrative review. DIGITAL HEALTH. (2025) 11:20552076251406654. doi: 10.1177/20552076251406654, 41393847 PMC12701246

[9] Teke J Olawade DB Leena N Weerasinghe K Mc Lernon S Moorley C. Digital health disparities: A review of barriers and solutions for racially diverse groups. Int J Med Inform. (2026) 206:106173. doi: 10.1016/j.ijmedinf.2025.106173, 41205583

[10] Du Y Yang P Liu Y Deng C Li X. Artificial intelligence in chronic disease self-management: current applications and future directions. Front Public Health. (2025) 13:1689911. doi: 10.3389/fpubh.2025.1689911, 41358250 PMC12675485

[11] Holmen H Fosse E. “I want to spend my time living”—experiences with a digital outpatient service with a Mobile app for tailored care among adults with Long-term health service needs: qualitative study using thematic analysis. J Med Internet Res. (2026) 28:e79155. doi: 10.2196/79155, 41538795 PMC12856408

[12] Rush KL Seaton CL Ross R Robertson T Louloudi A-I Loewen P . Digital literacy training within interventions to support older adults with cardiovascular disease in using technologies: A systematic review. Int J Med Inform. (2026) 211:106312. doi: 10.1016/j.ijmedinf.2026.106312, 41650700

[13] Zabczyk C Blakey JD. The effect of connected “smart” inhalers on medication adherence. Front Med Technol. (2021) 3:657321. doi: 10.3389/fmedt.2021.657321, 35047916 PMC8757760

[14] Pongpoottipatchara R Anonjarn K Prawatwong W Singha-Dong N. Implementation of digital device–assisted and nurse-led case management to promote self-management in adults with noncommunicable diseases: protocol for a single-arm intervention study. JMIR Res Protoc. (2026) 15:e80083. doi: 10.2196/80083 PMC12826159 41569800

[15] Robbins R Weaver MD Sullivan JP Quan SF Gilmore K Shaw S . Accuracy of three commercial wearable devices for sleep tracking in healthy adults. Sensors. (2024) 24:6532. doi: 10.3390/s24206532, 39460013 PMC11511193

[16] Stuart S De Kok M O’Searcoid B Morrisroe H Serban IB Jagers F . Critical design considerations for longer-term Wear and comfort of on-body medical devices. Bioengineering. (2024) 11:1058. doi: 10.3390/bioengineering11111058, 39593718 PMC11591458

[17] Wang M Lee K Thew HZ Sowtali SN Jiang Q Zhao Z . Effectiveness of the Mobile-based diabetes little helper video intervention on medication adherence among older adults living with type 2 diabetes mellitus, Henan, China: randomized controlled trial. J Med Internet Res. (2026) 28:e78731. doi: 10.2196/78731, 41701967 PMC12957950

[18] Palermo TM Ohls O Dear B Doorenbos AZ Yadav D Zhou C . Digital cognitive-behavioral therapy for pain Management in Individuals with recurrent acute and chronic pancreatitis (IMPACT-2): study protocol for a hybrid effectiveness-implementation trial. Trials. (2026) 27:204. doi: 10.1186/s13063-026-09517-6, 41664170 PMC12983583

[19] Jonassaint CR Lalama CM Carroll CP Badawy SM Hamm ME Stinson JN . Digital cognitive behavioral therapy vs education for pain in adults with sickle cell disease. Blood Adv. (2024) 8:6257–66. doi: 10.1182/bloodadvances.2024013861, 39374587 PMC11699089

[20] Huang H Liu F Mohammad M Watson R Hayter M Huang M . Digital gamification-based pursed lip breathing exercises driven by behaviour change wheel in patients with COPD: A feasibility trial protocol using pre-post study design. BMJ Open. (2025) 15:e090832. doi: 10.1136/bmjopen-2024-090832, 40147988 PMC11956336

[21] Roshan-Nejad M Hosseini M Vasli P Nasiri M Hejazi S. Effect of health literacy-based teach-Back training on quality of life and treatment adherence in type 2 diabetes: an experimental study. Sci Rep. (2025) 15:551. doi: 10.1038/s41598-024-84399-9, 39748080 PMC11696705

[22] Zhang Y Ngai FW Yang Q Xie YJ. Effectiveness of digital health interventions on sedentary behavior among patients with chronic diseases: systematic review and Meta-analysis. JMIR Mhealth Uhealth. (2025) 13:e59943–3. doi: 10.2196/59943, 40554804 PMC12212891

[23] Wang Z Li Y Wang Q Su Y. The effectiveness of wearable electronic device system–supported physical activity programs for cancer survivors: meta-analysis of randomized controlled trials. J Med Internet Res. (2025) 27:e74347. doi: 10.2196/74347 PMC12352708 40811695

[24] Dantu R Murad M Sharma K Dutta K Cravens-Ray L. Navigating illness in a virtual world: the role of immersive technology across chronic care continuum – A scoping review. Int J Med Inform. (2026) 211:106311. doi: 10.1016/j.ijmedinf.2026.106311, 41633285

[25] Ding ME Traiba H Perez HR. Virtual reality interventions and chronic pain: scoping review. J Med Internet Res. (2025) 27:e59922. doi: 10.2196/59922, 39965778 PMC11888090

[26] Belhan Z Muhan R Aksoy ME Ozker E Unver V. Examining the effect of virtual reality application on pain and anxiety in the Care of Chronic Wounds on the leg. J Tissue Viability. (2025) 34:100876. doi: 10.1016/j.jtv.2025.100876, 40037048

[27] Obrero-Gaitán E Chau-Cubero CY Lomas-Vega R Osuna-Pérez MC García-López H Cortés-Pérez I. Effectiveness of virtual reality-based therapy in pulmonary rehabilitation of chronic obstructive pulmonary disease. A systematic review with Meta-analysis. Heart Lung. (2024) 65:1–10. doi: 10.1016/j.hrtlng.2024.01.011, 38330853

[28] Du H Yu J Chen D Wu J Xue E Zhou Y . Big data–driven health portraits for personalized management in noncommunicable diseases: scoping review. J Med Internet Res. (2025) 27:e72636. doi: 10.2196/72636, 40472355 PMC12179573

[29] Shimbo D Shah RU Abdalla M Agarwal R Ahmad FS Anaya G . Transforming hypertension diagnosis and Management in the era of artificial intelligence: A 2023 National Heart, Lung, and Blood Institute (NHLBI) workshop report. Hypertension. (2025) 82:36–45. doi: 10.1161/HYPERTENSIONAHA.124.22095, 39011653 PMC11655265

[30] Liu Q Yang Y Hu Y Yang X Liu S Bao R . Study protocol for a randomized controlled trial to evaluate the effectiveness of an artificial intelligence-based health education accurately linking system based on traditional Chinese medicine body constitution in patients with chronic disease multimorbidity. Front Public Health. (2026) 14:1773974. doi: 10.3389/fpubh.2026.1773974, 41835430 PMC12982435

[31] Stefanelli A Zahia S Chanel G Niri R Pichon S Probst S. Developing an AI-powered wound assessment tool: A methodological approach to data collection and model optimization. BMC Med Inform Decis Mak. (2025) 25:297. doi: 10.1186/s12911-025-03144-y, 40783534 PMC12335118

[32] Joseph J. Algorithmic Bias in public health AI: A silent threat to equity in low-resource settings. Front Public Health. (2025) 13:1643180. doi: 10.3389/fpubh.2025.1643180, 40771228 PMC12325396

[33] Ruggiero C Waite M Stayt L. Experiences of remote nurse consultations in chronic disease management: A systematic review. Int J Nurs Stud. (2026) 175:105319. doi: 10.1016/j.ijnurstu.2025.105319, 41455203

[34] Colaone E Belluzzi E Pozzuoli A Roma E Bortolato E Biz C . Effectiveness of telemedicine nursing interventions in the Management of Overweight and Obesity in adults: A systematic review and Meta-analysis. Curr Obes Rep. (2025) 14:63. doi: 10.1007/s13679-025-00655-7, 40736661 PMC12310891

[35] Ruksakulpiwat S Pongsuwun K Junphongsri P Preeprem C Nguantad S Samart B. Nurse-led interventions to improve health, adherence, and functional outcomes in adults and older adults with multimorbidity: A systematic review of randomized and Quasiexperimental studies. J Nurs Manag. (2025) 2025:6252049. doi: 10.1155/jonm/6252049, 41019866 PMC12473742

[36] Periyakoil VS Von Gunten C Kraemer H. Virtual, nurse-led early primary palliative care intervention (ELICIT) for community-dwelling older adults with cognitive impairment: protocol for a randomized controlled trial. JMIR Res Protoc. (2025) 14:e75082–2. doi: 10.2196/75082, 41442238 PMC12731548

[37] Azzellino G Passamonti M Aitella E Mengoli L Vagnarelli P Ginaldi L . Bridging the gap in chronic disease management: a nursing perspective on the use of predictive tools and telemedicine in the hospital–community transition. Medicina (B Aires). (2025) 61:2213. doi: 10.3390/medicina61122213 PMC12735139 41470216

[38] March CA Rothenberger S Naame E Stouffer V Moon C Lyon AR . School-partnered collaborative care intervention for young children with type 1 diabetes: A pilot and feasibility protocol. Contemp Clin Trials. (2026) 163:108259. doi: 10.1016/j.cct.2026.108259, 41707755 PMC13004637

[39] Pires MDCCM Barbieri - Figueiredo MDCA Cardoso DFB Duque FM Tricas-Sauras MS Prosen M . Technology-mediated training programs for school health teams on special health care needs: a scoping review. Rev Esc Enferm USP. (2025) 59:e20240328. doi: 10.1590/1980-220x-reeusp-2024-0328en, 40341907 PMC12064086

[40] German J Yang Q Hatch D Lewinski A Bosworth HB Kaufman BG . EXpanding technology-enabled, nurse-delivered chronic disease care (EXTEND): protocol and baseline data for a randomized trial. Contemp Clin Trials. (2024) 146:107673. doi: 10.1016/j.cct.2024.107673, 39216685 PMC11531378

[41] Huang X Song X Sun M Hou Y Nan J Gao J . Effectiveness of digital co-creation platform in remote pulmonary rehabilitation for older adults with chronic obstructive pulmonary disease: A randomized controlled trial. Front Public Health. (2025) 13:1708607. doi: 10.3389/fpubh.2025.1708607, 41293596 PMC12640875

[42] Steiner B Verket M Baldewijns K Murphy M McNulty A Schwertfeger M . Participative research for individualised Care in Cardiovascular Diseases (PRIC-CVD): study protocol for a non-interventional, multicentre mixed-methods study as part of iCARE4CVD. BMJ Open. (2025) 15:e098061. doi: 10.1136/bmjopen-2024-098061, 40615140 PMC12226951

[43] Clifford N Tunis R Ariyo A Yu H Rhee H Radhakrishnan K. Trends and gaps in digital precision hypertension management: scoping review. J Med Internet Res. (2025) 27:e59841. doi: 10.2196/59841, 39928934 PMC11851032

[44] Kelley MM Powell T Camara D Shah N Norton JM Deitelzweig C . Mobile health apps, family caregivers, and care planning: scoping review. J Med Internet Res. (2024) 26:e46108. doi: 10.2196/46108, 38781588 PMC11157180

[45] Riegel B Quinn R Hirschman KB Thomas G Ashare R Stawnychy MA . Health coaching improves outcomes of informal caregivers of adults with chronic heart failure: a randomized controlled trial. Circ Heart Fail. (2024) 17:e011475. doi: 10.1161/CIRCHEARTFAILURE.123.011475, 38904103 PMC11244754

[46] Shankar R Hong WZ Bundele A Si KP Mukhopadhyay A. Virtual reality-guided mindfulness intervention to enhance psychosocial well-being in caregivers of end-stage kidney disease patients: protocol for a mixed-methods pilot randomised controlled trial. BMJ Open. (2024) 14:e090550. doi: 10.1136/bmjopen-2024-090550, 39438104 PMC11499788

[47] Wang J Li Q Cui J Tu S Deng Z Yang R . Effectiveness of virtual reality on the caregiving competence and empathy of caregivers for elderly with chronic diseases: A systematic review and Meta-analysis. J Nurs Manag. (2023) 2023:1–15. doi: 10.1155/2023/5449955, 40225657 PMC11918966

[48] Palermo TM Srinakarin K Zhou C Lalloo C Dampier C Zempsky WT . Moderators of digital cognitive-behavioral therapy for youth with sickle cell disease pain: secondary analysis of a randomized controlled trial. Pain. (2025) 166:e233–43. doi: 10.1097/j.pain.0000000000003583, 40839690 PMC12353042

[49] Ho HT Jenkins C Ta HQ Bui CL Van Hoang M Santin O. Digital support for caregivers of patients with non-communicable diseases during COVID-19: lessons from a Cancer case study in Vietnam. J Glob Health. (2021) 11:03095. doi: 10.7189/jogh.11.03095, 34408856 PMC8364252

[50] Hwang M Lee S Park GE Park Y-H. Effectiveness of a digital health coaching self-management program for older adults living alone with multiple chronic conditions: A randomized controlled trial. Geriatr Nurs. (2025) 65:103509. doi: 10.1016/j.gerinurse.2025.103509, 40633238

[51] Sheng Y Bond R Jaiswal R Dinsmore J Doyle J. Augmenting K-means clustering with qualitative data to discover the engagement patterns of older adults with multimorbidity when using digital health technologies: proof-of-concept trial. J Med Internet Res. (2024) 26:e46287. doi: 10.2196/46287, 38546724 PMC11009852

[52] Kisa S Kisa A. Can digital storytelling improve health outcomes for immigrant and refugee populations? A scoping review. BMC Public Health. (2025) 25:1043. doi: 10.1186/s12889-025-22209-1, 40102838 PMC11916951

[53] Zhang K Huang Q Wang Q Li C Zheng Q Li Z . Telemedicine in improving glycemic control among children and adolescents with type 1 diabetes mellitus: systematic review and Meta-analysis. J Med Internet Res. (2024) 26:e51538. doi: 10.2196/51538, 38981114 PMC11267117

[54] Hommel KA Robbertz A Kabbouche M LeCates S Ritterband LM Peugh J . Protocol for a randomized controlled trial evaluating a digital behavioral self-management intervention for adolescent migraine. Appl Nurs Res. (2025) 84:151979. doi: 10.1016/j.apnr.2025.151979, 40744542 PMC12954598

[55] Li Z Lu F Wu J Bao R Rao Y Yang Y . Usability and effectiveness of eHealth and mHealth interventions that support self-management and health care transition in adolescents and young adults with chronic disease: systematic review. J Med Internet Res. (2024) 26:e56556. doi: 10.2196/56556, 39589770 PMC11632288

[56] Yao L Li Q Liu Y Li Q Wang T Zhou Z . How to assess multimorbidity: A systematic review. Front Public Health. (2025) 13:1525593. doi: 10.3389/fpubh.2025.1525593, 40213437 PMC11983592

[57] Ogungbe O Jabakhanji SB Mehta R McCaffrey J Byrne D Hurley S . Disruption to diabetes and hypertension care during the COVID-19 pandemic in Latin America and the Caribbean and mitigation approaches: A scoping review. BMC Health Serv Res. (2025) 25:660. doi: 10.1186/s12913-025-12760-3, 40341010 PMC12060407

[58] Karan A Hussain S Jensen LX Buhl A Bearman M Zodpey S. Non-communicable diseases, digital education and considerations for the Indian context – a scoping review. BMC Public Health. (2024) 24:1280. doi: 10.1186/s12889-024-18765-7, 38730455 PMC11083803

[59] Shegal M Hu LT Friesen E Minian N Maslej M Rodak T . Conversational agent interventions in diabetes care: A systematic review. Diabetes Res Clin Pract. (2025) 228:112429. doi: 10.1016/j.diabres.2025.112429, 40840786

[60] Zhou J Ding S Xu Y Pan H. Effects of digital intelligent interventions on self-Management of Patients with Diabetic Foot: systematic review. J Med Internet Res. (2025) 27:e64400. doi: 10.2196/64400, 40132178 PMC11979535

[61] Zhuang M Hassan II W Ahmad WMA Abdul Kadir A Liu X Li F . Effectiveness of digital health interventions for chronic obstructive pulmonary disease: systematic review and meta-analysis. J Med Internet Res. (2025) 27:e76323. doi: 10.2196/76323, 40418567 PMC12149779

[62] Wang S-C Kuo T-H Rai C-I Chen Y-C. Inhalation: a smart strategy and increasing potential for drug delivery. DDDT. (2026) 20:1–16. doi: 10.2147/DDDT.S575392, 41710582 PMC12912003

[63] Pittara M Matsangidou M Pattichis CS. Virtual reality for pulmonary rehabilitation: comprehensive review. JMIR Rehabil Assist Technol. (2023) 10:e47114. doi: 10.2196/47114 PMC10580136 37782529

[64] O’Connor Ú Crilly G. The experiences and perceptions of telehealth in patients living with advanced chronic obstructive pulmonary disease: A qualitative evidence synthesis. J Adv Nurs. (2025) 81:1700–16. doi: 10.1111/jan.16493, 39420778 PMC11896827

[65] Boyers D Cruickshank M Azharuddin M Manson P Swallow D Counsell C . Digitally enabled therapy for chronic tic disorders and Tourette syndrome: A systematic review and economic evaluation. Health Technol Assess. (2026) 30:1–67. doi: 10.3310/QLAS8524, 41631683 PMC12884836

[66] Matthie N Higgins M Doorenbos A Maddox T Jenerette C. Feasibility of in-home virtual reality for chronic pain in sickle cell disease. Pain Manag Nurs. (2024) 25:425–31. doi: 10.1016/j.pmn.2024.04.007, 38697889 PMC11324415

[67] Moss-Morris R Norton C Hart A Cléirigh Büttner F Hamborg T Miller L . Digital cognitive behavioural self-management programme for fatigue, pain, and faecal incontinence in inflammatory bowel disease (IBD-BOOST): a multicentre, parallel, randomised controlled trial. Lancet Digit Health. (2025) 7:100906. doi: 10.1016/j.landig.2025.100906, 41206261

[68] Thai YC Sim D McCaffrey TA Ramadas A Malini H Watterson JL. A scoping review of digital workplace wellness interventions in low- and middle-income countries. PLoS One. (2023) 18:e0282118. doi: 10.1371/journal.pone.0282118, 36854022 PMC9974126

[69] De Souza Ferreira E De Aguiar Franco F Dos Santos Lara MM Levcovitz AA Dias MA Moreira TR . The effectiveness of Mobile application for monitoring diabetes mellitus and hypertension in the adult and elderly population: systematic review and Meta-analysis. BMC Health Serv Res. (2023) 23:855. doi: 10.1186/s12913-023-09879-6, 37573312 PMC10423411

[70] Jiang R Chang G Liu T Xu L Zhang X Zhou Y . National and province-level primary health care policies for the prevention and control of non-communicable diseases in China from 2009 to 2023: A scoping review. BMJ Open. (2025) 15:e097826. doi: 10.1136/bmjopen-2024-097826, 40280618 PMC12035461

